# Pu(iii) and Cm(iii) in the presence of EDTA: aqueous speciation, redox behavior, and the impact of Ca(ii)†

**DOI:** 10.1039/d1ra09010k

**Published:** 2022-03-25

**Authors:** Nicole A. DiBlasi, Agost G. Tasi, Michael Trumm, Andreas Schnurr, Xavier Gaona, David Fellhauer, Kathy Dardenne, Jörg Rothe, Donald T. Reed, Amy E. Hixon, Marcus Altmaier

**Affiliations:** Department of Civil & Environmental Engineering & Earth Sciences, University of Notre Dame 301 Stinson-Remick, Notre Dame IN 46556 USA hixon@nd.edu nicole.a.diblasi@gmail.com; Karlsruhe Institute of Technology, Institute for Nuclear Waste Disposal P.O. Box 3640 Karlsruhe 76021 Germany xavier.gaona@kit.edu; Los Alamos National Laboratory 1400 University Dr. Carlsbad NM 88220 USA

## Abstract

The impact of calcium on the solubility, redox behavior, and speciation of the An(iii)–EDTA (An = Pu or Cm) system under reducing, anoxic conditions was investigated through batch solubility experiments, X-ray absorption spectroscopy (XAS), density functional theory (DFT), and time-resolved laser fluorescence spectroscopy (TRLFS). Batch solubility experiments were conducted from undersaturation using Pu(OH)_3_(am) as the solid phase in contact with 0.1 M NaCl–NaOH–HCl–EDTA–CaCl_2_ solutions at [EDTA] = 1 mM, pH_m_ = 7.5–9.5, and [CaCl_2_] ≤20 mM. Additional samples targeted brine systems represented by 3.5 M CaCl_2_ and WIPP simulated brine. Solubility data in the absence of calcium were well-described by Pu(iii)–EDTA thermodynamic models, thus supporting the stabilization of Pu(iii)–EDTA complexes in solution. Cm(iii)–EDTA TRLFS data suggested the stepwise hydrolysis of An(iii)-EDTA complexes with increasing pH, and current Pu(iii)-EDTA solubility models were reassessed to evaluate the possibility of including Pu(iii)–OH–EDTA complexes and to calculate preliminary formation constants. Solubility data in the presence of calcium exhibited nearly constant log *m*(Pu)_tot_, as limited by total ligand concentration, with increasing [CaCl_2_]_tot_, which supports the formation of calcium-stabilized Pu(iii)–EDTA complexes in solution. XAS spectra without calcium showed partial oxidation of Pu(iii) to Pu(iv) in the aqueous phase, while calcium-containing experiments exhibited only Pu(iii), suggesting that Ca–Pu(iii)–EDTA complexes may stabilize Pu(iii) over short timeframes (*t* ≤45 days). DFT calculations on the Ca–Pu(iii)–EDTA system and TRLFS studies on the analogous Ca–Cm(iii)–EDTA system show that calcium likely stabilizes An(iii)–EDTA complexes but can also potentially stabilize An(iii)–OH–EDTA species in solution. This hints towards the possible existence of four major complex types within Ca–An(iii)–EDTA systems: An(iii)–EDTA, An(iii)–OH–EDTA, Ca–An(iii)–EDTA, and Ca–An(iii)–OH–EDTA. While the exact stoichiometry and degree of ligand protonation within these complexes remain undefined, their formation must be accounted for to properly assess the fate and transport of plutonium under conditions relevant to nuclear waste disposal.

## Abstract 2

Combined advanced spectroscopy and solubility studies provide evidence for the formation of novel calcium-containing and hydrolyzed (Cm,Pu)(iii)–EDTA complex(es).

## Graphical abstract

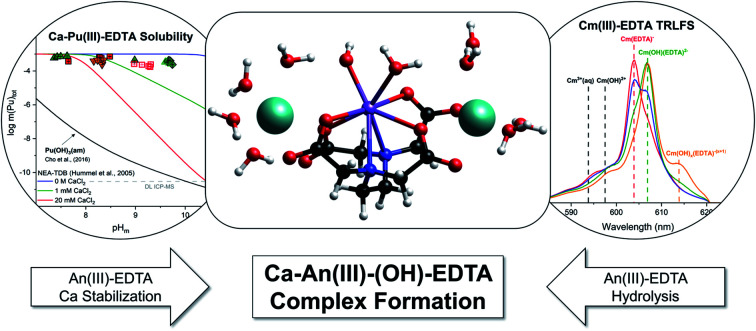

## 1. Introduction

Deep geologic repositories for nuclear waste disposal utilize natural and engineered barriers to prevent the release of radionuclides into the geosphere, and many of these barriers contribute to establishing boundary conditions.^1,2^ Deep geologic repositories are expected to develop strongly reducing, anoxic environments post-closure due to the corrosion of iron and steel. Alkaline (pH ≈ 9) to hyperalkaline (pH ≤13.3) buffered pH is also expected due to the presence of engineered barrier materials, such as MgO and/or cement, respectively.^3,4^ These conditions strongly affect the chemical behavior of radionuclides disposed of within a repository.

A comprehensive understanding of radionuclide interactions and processes that may occur in the case of water intrusion in a deep geologic repository is necessary for long-term safety assessments, development of the Safety Case, and accurately predicting radionuclide fate and transport.^5^ One primary radionuclide of concern is plutonium because of its prevalence in both commercial, spent nuclear fuel and transuranic waste considering its high alpha radiotoxicity and long half-life (*e.g.*, *t*_1/2_^239^Pu = 2.41 × 10^4^ a).

Ethylenediaminetetraacetic acid (EDTA, C_10_H_16_N_2_O_8_) is an organic molecule known to strongly chelate metal ions in aqueous solution. EDTA has been co-disposed with plutonium in many waste streams, and is expected to be present in deep geologic repository concepts due to its extensive use as a decontamination agent and in the processing of plutonium-containing wastes.^6–9^ The presence of EDTA in these wastes can enhance the solubility of plutonium *via* the formation of stable aqueous complexes; thus, it can substantially increase the environmental mobility of plutonium by outcompeting its sorption and other retarding processes.^9–13^

Plutonium has four relevant oxidation states (iii–VI) in aqueous environmental systems. As repositories are expected to have alkaline and strongly reducing conditions post-closure, Pu(iii) must be considered in repository models. For example, the Waste Isolation Pilot Plant (WIPP) in Carlsbad, New Mexico (USA), the only currently-operating deep geologic repository in the United States of America, assumes a 50% distribution of both Pu(iii) and Pu(iv) within their repository concept.^4^ Pu(iii) can be reasonably stable in reducing, aqueous solutions and is considered quite soluble in comparison to Pu(iv).^14^

EDTA has been shown to exhibit preferential complexation with plutonium in the +iv oxidation state. However, recent studies show that Pu(iii) persists in the presence of EDTA up to 237 days and that PuO_2_(ncr,hyd) solubility in the presence of EDTA can potentially lead to the formation of aqueous Pu(iii)-EDTA complexes.^9,11,12^ For these reasons, it is essential to dedicate experimental efforts for the characterization of Pu(iii) interactions with the expected organic ligands within repository concepts. To further investigate these interactions, analogous studies with curium can be used to probe plutonium complexation in the +iii oxidation state due to the similar effective ionic radii (*r*_Cm(III)_ = 0.97 Å; *r*_Pu(III)_ = 1.00 Å; both with a coordination number of 6) and chemical characteristics of the two elements.^15–17^

Calcium is ubiquitous in environmental systems and host rock formations, and thus is also expected in geologic repositories. Cementitious materials can be a source of calcium in some repository concepts, primarily as calcium silicate hydrate (C–S–H) phases and portlandite (Ca(OH)_2_).^18–20^ The corrosion of cementitious waste forms in the presence of concentrated MgCl_2_ solutions can also generate CaCl_2_-rich brines (up to 4 M) with high pH values (pH_m_ ≈ 12).^3^ Calcium is known to form a predominantly stable complex with EDTA (log *β*° = (12.690 ± 0.060)),^21^ but it can also stabilize negatively-charged, M–L complexes (M = metal, L = ligand) *via* formation of stable ternary Ca–M–L or quaternary Ca–M–OH–L species.

Our recent study utilized a combination of undersaturation solubility studies using a thoroughly characterized PuO_2_(ncr,hyd) solid phase with a well-defined solubility product and solubility data from the literature to generate a comprehensive thermodynamic description of the Pu(iv)–EDTA system under conditions relevant for nuclear waste disposal.^12^ Our results support the formation of quaternary Ca–Pu(iv)–OH–EDTA complex(es) over a wide range of total calcium concentrations (1 mM ≤ [Ca(ii)]_tot_ ≤ 3.5 M), which are similar to complexes observed and described for lanthanides and actinides with other complexing agents like gluconate (C_6_H_12_O_7_), isosaccharinic acid (C_6_H_12_O_6_), and citrate (C_6_H_8_O_7_).^22–27^

In the present work, we investigated the solubility, complexation, and redox behavior of An(iii) (An = Pu or Cm) in the presence of EDTA and calcium. Plutonium experiments were performed under controlled redox conditions using a combination of solubility experiments and advanced spectroscopic techniques. Laser fluorescence spectroscopy of curium was used to further probe An(iii)–EDTA speciation. Special focus was given to the role of both hydrolysis and calcium in these systems, which can either act as competitors with plutonium for complexation of EDTA or as components contributing to the formation of stable ternary or quaternary complexes. Using our own experimental data and computational modeling, this work aims to describe An(iii)–EDTA speciation under conditions relevant for nuclear waste disposal.

## 2. Thermodynamic background

The speciation of Pu(iii) in the presence of EDTA is extensively studied within the literature. A comprehensive critical review of thermodynamic studies of systems relevant for nuclear waste disposal was published in 2005 by the Nuclear Energy Agency Thermochemical Database project (NEA-TDB).^21^ This reviewed nearly all available literature on the Pu(iii)–EDTA system dating back to the late 1950s and analyzed it for selection within the database project based on strict criteria.^28–36^ From this review process, two Pu(iii)–EDTA complexes were selected within the NEA-TDB with log *β*°(Pu(EDTA)^−^) = (20.180 ± 0.370) and log *β*°(Pu(HEDTA)(aq)) = (22.020 ± 0.260). Both selected constants were taken from Merciny *et al.*,^32^ who utilized potentiometric titrations at pH = 1–9.5 and *I* = 1 M KCl to investigate the Pu(iii)–EDTA system. The authors identified 6 different aqueous Pu(iii)–EDTA species: Pu(EDTA)^−^, Pu(HEDTA)(aq), Pu(EDTA)_2_^5−^, Pu(EDTA)_2_H^4−^, Pu(EDTA)_2_H_2_^3−^, and Pu(EDTA)_2_H_3_^2−^; the first two species were identified through a series of titrations from pH 1.56–2.39, whereas the latter four species were identified at higher pH and exhibited significantly weaker complexation. The NEA-TDB did not select the latter four species because “*in view of the weak complexation effects, the ambiguities involved in extrapolating highly charged species from 1 M KCl to zero ionic strength using estimated SIT interaction parameters, and the fact that no other reliable study has yet confirmed the results of [Merciny et al.*^32^*], no values are selected in this review for 1:2 complexes”*.^21^

More recently, Rai *et al.*^37^ used solubility studies to probe Pu(iii)–EDTA speciation. The authors performed a series of systematic solubility studies with PuPO_4_(cr,hyd) solid phase under four distinct sets of conditions: (i) varied time and pH (1–12) with fixed phosphate concentration (0.00032 M), (ii) varied phosphate concentrations (0.0001–1.0 M) at pH = 2.5, (iii) varied time and pH (1.3–13) with fixed phosphate (0.00032 M) and EDTA concentrations (0.0004 or 0.002 M), and (iv) varied EDTA concentrations (0.00005–0.0256 M) with fixed phosphate concentration (0.00032 M) and pH (3.5, 10.6, or 12.6). The solid phase used by Rai *et al.*,^37^ synthesized using ^239^Pu, was extensively characterized and the solubility product was determined as log *K*_s,0_° = –(24.28 ± 0.35). The redox conditions were controlled using either hydroquinone or sodium dithionite as redox buffers. The authors used their solubility data points after ∼75 days of equilibration to derive thermodynamic and activity models, which consist of one predominant Pu(iii)–EDTA species with only log *β*°(Pu(EDTA)^−^) = (20.15 ± 0.59) needed for their data interpretation. Both Pitzer and the specific ion-interaction theory (SIT) activity models, which consider interactions between all ions present in solution or only interactions between charged ions, respectively,^38,39^ were derived and implemented by Rai *et al.*^37^ for ionic strength corrections within their work.

Although curium complexes are not currently included in the organic complexation selection of the NEA-TDB, Cm–EDTA complexation has been investigated since the late 1950s.^40–52^ A recent study by Thakur *et al.*^51^ investigated Cm–EDTA complexation as a function of ionic strength (0.1–6.60 m NaClO_4_) using solvent extraction at pH 3.6 and potentiometric titration from pH 2–11. The authors applied Pitzer ionic strength corrections to generate a formation constant for the Cm(EDTA)^−^ complex (log *β*° = (20.43 ± 0.20)). The formation of a hydrolyzed complex, Cm(OH)(EDTA)^2−^, has also been proposed *via* spectrophotometry, fluorescence, and luminescence spectroscopy.^44,52^ Specifically, Griffiths *et al.*^44^ observed that at a 1 : 1 : 1 An(iii) : EDTA : CO_3_^2−^ ratio and a pH ≥10 hydroxide was more likely to replace carbonate within ternary An(iii)–CO_3_–EDTA complex(es) in preference to replacing the EDTA^4−^, thus emphasizing the affinity of OH^−^ groups for coordination with An(iii)–EDTA complex(es) at elevated pH. The authors also highlighted that no precipitates were observed up to a pH of 11, illustrating the strong binding affinity of EDTA^4−^ to trivalent actinides and the stability of the proposed hydrolyzed species under hyperalkaline pH conditions. Using a comparison to literature data and a non-linear free energy relationship, Hummel also proposed the existence of the hydrolyzed EDTA species An^III^(OH)(EDTA)^2−^ (with An = Np, Pu, Am and Cm), and provided estimates for the corresponding formation constants.^53^ Additionally, a value is provided within the NIST Database 46 for the equilibrium constant of the complex Pu^III^(OH)(EDTA)^2−^, although we have not been able to trace these data back to any experimental study available in the literature.^48^ As M(iii)–OH–EDTA complexes have been observed for both lanthanides (*e.g.*, Eu)^40,52,54–56^ and actinides (*e.g.*, Am),^44,57,58^ the existence of Pu(iii)–OH–EDTA aqueous complex(es) can be postulated even though they have not yet been investigated experimentally.

The NEA-TDB selects two Ca–EDTA species—log *β*°(Ca(EDTA)^2−^) = (12.690 ± 0.060) and log *β*°(Ca(HEDTA)^−^) = (16.230 ± 0.108)—and both complexes are considered in the thermodynamic calculations in the presence of calcium within this work.^21^ Further discussion of Ca–EDTA formation constant selection can be found elsewhere.^12,21^ The great stability of Ca–EDTA complexes has led to the general assumption that the presence of calcium will largely outcompete the complexation of EDTA with other metal ions present in significantly lower concentrations.

## 3. Experimental

### 3.1 Materials

Experiments with plutonium were performed in the controlled area of KIT-INE. All experiments were conducted in argon gloveboxes with O_2_ <2 ppm and under carbonate exclusion. All experimental solutions were prepared with ultra-pure water purified with a Milli-Q apparatus (Millipore, 18.2 MΩ cm, 22 ± 2 °C). Before use, Milli-Q water was boiled for several hours while being purged with argon gas. EDTA stock solutions were prepared from sodium salts (Sigma Aldrich) to target specific pH values: H_4_EDTA (purified grade ≥98.5%), Na_2_H_2_EDTA (99.0–101.0%), Na_3_HEDTA·*x*H_2_O (≥95%), and Na_4_EDTA·2H_2_O (99.0–101.0%). Calcium-containing solutions were prepared with CaCl_2_·2H_2_O (Millipore). Redox buffers were prepared using Na_2_S_2_O_4_ (Merck, ≥87%, hereafter denoted as DT) and SnCl_2_ (Sigma Aldrich, reagent grade 98%, hereafter denoted as Sn(ii)). TRIS buffer solutions were prepared with pre-determined ratios of Trizma® base and Trizma® hydrochloride, as purchased (Sigma Aldrich). Ionic strength was kept constant at *I* = 0.1 M using NaCl (p.a.), except in samples containing 3.5 M CaCl_2_ and samples prepared in the WIPP simulated brine. The NaOH and HCl solutions used for pH adjustments were prepared from standard solutions (Merck, Titrisol®). Plutonium solids were prepared from a plutonium stock in 2.0 M HClO_4_ with an isotopic composition of 99.4% ^242^Pu, 0.58% ^239^Pu, 0.005% ^238^Pu, and 0.005% ^241^Pu. Curium experimental solutions were prepared from a 10^−5^ M curium stock in 0.001–0.01 M HCl with an isotopic composition of 89.68% ^248^Cm, 9.38% ^246^Cm, 0.43% ^243^Cm, 0.30% ^244^Cm, 0.14% ^245^Cm and 0.07% ^247^Cm.

### 3.2 Preparation of plutonium solid phases

Pu(OH)_3_(am) was freshly synthesized for use in the current work (see ESI†). Briefly, a Pu(iv) stock solution in 2 M HClO_4_ was electrochemically reduced to Pu(iii)_aq_. The resulting Pu(iii) stock solution was subsequently precipitated as Pu(OH)_3_(am) through slow addition to a TRIS buffer solution (pH_m_ = 9.47) containing DT to maintain reducing conditions. Following precipitation, the solid phase was washed with and subsequently suspended in a pH 11 NaOH solution. The resulting solid phase was used as reference material in the X-ray absorption spectroscopy study and added to solubility experiments within one day of preparation.

Details on the synthesis procedure and the characterization results of the nanocrystalline PuO_2_(ncr,hyd) solid phase used as a reference material in the current X-ray absorption near edge structure (XANES) study are published elsewhere.^59^ By the initialization of these experiments, the PuO_2_(ncr,hyd) phase had aged *ca*. 11 years in 0.1 M NaCl–HCl media under argon atmosphere.

### 3.3 Time resolved laser fluorescence spectroscopy experiments

Curium was used as a fluorescence probe within this work. Time resolved laser fluorescence spectroscopy (TRLFS) studies were performed as a series of titrations as a function of calcium concentration (0 M ≤ [CaCl_2_]_tot_ ≤ 3.5 M) at constant pH_m_ (7, 9, 11 or 12), curium concentration (10^−7^ M), EDTA concentration (1 mM), and ionic strength (*I* = 0.1 or 10.5 M). After each progressive titration, the pH of the solution was adjusted using the appropriate concentration of HCl or NaOH and allowed to equilibrate for 24 hours before TRLFS analysis.

For TRLFS measurements, a pulsed Nd : YAG (Continuum Surelite II) pumped dye laser system (Radiant dyes Narrow Scan, repetition rate: 10 s^−1^) was operated at a constant excitation wavelength of *λ* = 396.6 nm and a pulse energy between 2 and 4 mJ with the dye Exalite 398. The optical multichannel analyzer consisted of an ICCD-camera (iStar, Andor) and a polychromator (Shamrock 303i, Andor) with a 1200 lines per mm grating and a spectral range of 580–620 nm. The spectra were measured 1 μs after laser pulse in a time window of 1 ms. Fluorescence lifetime measurements were performed by monitoring the fluorescence emission as a function of the delay time between laser pulse and camera grating with delay time steps of 10–40 μs. Fluorescence emission lifetime (*τ*) was obtained by fitting both the integrated intensity (*I*) and the absolute intensity at peak maxima positions as a function of delay time (*t*) following the equation *I*(*λ*) = *I*_0_(*λ*) × *e*^(−*t*/*τ*)^, where *I*_0_ is the intensity at *t* = 0. The number of coordinated water molecules (*N*_H_2_O_) was calculated using eqn (1) as reported in Kimura *et al.*^60^ where *k*_obs_ is the fluorescence decay constant.

1 *N*_H_2_O_ = 0.65*k*_obs_ − 0.88

### 3.4 Solubility experiments

Batch-type undersaturation solubility experiments using freshly precipitated Pu(OH)_3_(am) were carried out as a function of CaCl_2_ concentration (0 M ≤ [CaCl_2_]_tot_ ≤ 3.5 M) at constant pH_m_ = 9 and [EDTA] = 1 mM. Table S3† details the experimental conditions for each individual batch experiment. The ionic strength of all solutions was kept constant at 0.10 M accounting for the contribution of all the components: Na_4_EDTA–Na_3_HEDTA–Na_2_H_2_EDTA–NaCl–HCl–NaOH–CaCl_2_. Exceptions were the samples with the highest calcium concentration ([CaCl_2_] = 3.5 M, *I* = 10.5 M) and the samples prepared in the WIPP simulated brine (see Table S4† for brine formulation). The pH_m_ of each sample was adjusted with the appropriate concentration of HCl and NaOH to maintain constant ionic strength, whereas reducing conditions were kept with either DT or Sn(ii). Pu(OH)_3_(am) was added to experiments through the following procedure: aliquots of a Pu(OH)_3_(am) suspension (in pH 11 NaOH) were added to microcentrifuge tubes and compacted through centrifugation (6000 rpm for 5–10 minutes, ∼2000 g), the supernatant was removed, the solid phase was resuspended in the experimental solution and added to the batch reactor. Following Pu(OH)_3_(am) addition, pH_m_, *E*_h_, and aqueous plutonium concentrations (after 10 kD ultrafiltration) were monitored up to 45 days.

### 3.5 Solution characterization analytical methods

All pH measurements were performed using a combination pH electrode (type Orion Ross, Thermo Scientific™) freshly calibrated against standard pH buffers (pH = 3–13, Merck). To account for any ionic strength effects, we report pH as pH_m_ (pH_m_ = −log *m*(H^+^) = pH_exp_ + *A*_m_), or the total free concentration of protons in molal units. In aqueous solutions of ionic strength *I* ≥0.1 M, the measured pH value (pH_exp_) is an operational, apparent value related to *m*(H^+^) by *A*_m_, an empirical parameter that includes the activity coefficient of the proton (*γ*H^+^) and the liquid junction potential of the electrode for a given background electrolyte, ionic strength, temperature, and pressure. Empirical *A*_m_ values for NaCl and CaCl_2_ systems were adapted from the literature.^61,62^

The redox potential in solution was determined with combined Pt, Ag/AgCl reference electrodes (Metrohm). The measured potentials were converted to *E*_h_ through a standard correction for the potential of the Ag/AgCl inner-reference electrode at 3 M KCl and *T* = 22 °C (+207 mV). *E*_h_ values were further converted to pe (pe = −log *a*_e_^−^) by eqn (2):

2 *E*_h_ = −*RT* ln(10)*F*^−1^ log *a*_e_^−^

where *R* is the ideal gas constant (8.31446 J mol^−1^ K^−1^), *F* is the Faraday constant (96 485.33 C mol^−1^), and *a*_e_^−^ is the activity of the electron. Each redox potential measurement was allowed a minimum of 15 minutes for equilibration until the absolute drift of the value was observed to be below 3.0 mV min^−1^. Systematic increases in experimentally-measured pe values were observed over time, which are attributed to the sorption of EDTA, Sn(ii), and/or DT onto the surface of the *E*_h_ probe. Hour-long *E*_h_ measurements were performed after cleaning the electrode with 1.0 M HCl to verify the validity of the values obtained through the 15 minute measurements. The hour-long acquisitions resulted in an absolute electrode drift of ≤0.5 mV min^−1^ and values that were equivalent to initial pe measurement values. Overall uncertainties of measured *E*_h_ values (calculated as 2*σ* of repeated measurements) ranged between ±15 and ±40 mV.

The total molar aqueous plutonium concentration ([Pu]_tot_) was quantified after phase separation using quadrupole inductively coupled plasma mass spectrometry (ICP-MS, PerkinElmer™ NexION® 2000) and/or sector-field ICP-MS (SF-ICP-MS, Thermo Scientific™ ELEMENT™). Phase separation was achieved on an aliquot of the original sample through 10 kD centrifugal filters (pore size ≈ 2–3 nm, Nanosep®, Pall Life Sciences) at 6000 rpm (∼2000 g) for 15 minutes. The filtrates were directly diluted in 2% HNO_3_ before analysis. Molar concentrations (mol L^−1^, [Pu]_tot_) were converted to molal units (mol kg_w_^−1^, *m*(Pu)_tot_) using conversion factors reported elsewhere.^14^

### 3.6 X-ray absorption spectroscopy

X-ray absorption spectroscopy (XAS) analyses of selected aqueous and solid phases were recorded at the INE-Beamline for Actinide Research at the KIT synchrotron light source, KIT Campus NORD.^63,64^ The storage ring operated at 2.5 GeV electron energy with a mean electron current of 120 mA. Solid phases were characterized by X-ray absorption near edge structure (XANES) analyses to gain insight on the plutonium solid phase controlling the solubility in undersaturation experiments. Aqueous phases were characterized by XANES to experimentally determine the oxidation state distribution of plutonium in solution.

For the purpose of these analyses, selected plutonium samples were transferred into polyethylene vials under an argon atmosphere. A suspension of approximately 1 mg of the solid material was pipetted into the vial, tightly sealed with Parafilm® (Bemis Company, Inc.) and centrifuged for a minimum of 10 minutes at 6000 rpm (∼2000 g) to compact the solid into the bottom. Once the solid was sufficiently compacted, this sample vial was used for both aqueous and solid phase analyses. This method allowed for the collection of XAS measurements without disturbing the system equilibrium. Following solid compaction, the vials were mounted with the use of Kapton® tape into a gas-tight cell within an argon glovebox and transported to the INE-Beamline. During measurements, argon was continuously flushed through the cell ensuring the presence of an inert atmosphere.

XAS spectra of the Pu L_III_-edge (18 057 eV) were recorded in fluorescence detection mode using a combination of two Silicon Drift Detectors (SDD)—a Vortex®-ME4 (4 elements) and a Vortex-60EX (1 element) (Hitachi/SIINT, both 1 mm crystal thickness). Incident beam intensity and the transmission of a reference 20 μm zirconium metal foil were recorded simultaneously using argon-filled ionization chambers at ambient pressure; 3–9 scans were collected for each sample.

XANES data reduction was performed with the ATHENA and ARTEMIS software from the Demeter 0.9.26 program package^65^ following standard procedures. The Pu L_III_-edge spectra obtained in this work were calibrated against the first inflection point in the K-edge spectrum of the zirconium metal foil (K-edge = 17 998 eV) and averaged to reduce statistical noise. *E*_0_ for the Pu L_III_-edge was selected at the white line maxima. The spectra were then compared with Pu(iii)_aq_, Pu(iv)_aq_, Pu(OH)_3_(am), and PuO_2_(ncr,hyd) reference spectra collected at the INE-Beamline under similar experimental conditions and data analysis procedures.^59,66^

### 3.7 Data analysis and modeling methods

The thermodynamic calculations in this work are based on the reactions and associated constants summarized in Section 2 and provided in Table S1.† The specific ion-interaction theory (SIT) was used for ionic strength corrections,^38^ and the ion–interaction parameters (Table S2†) were either taken from the literature^14,21,67–69^ or estimated based on the charge correlation approach described by Hummel.^70^ The PHREEPLOT-PHREEQC Interactive software package was applied for solubility calculations and data analysis/modeling (version 3.4.0, svn 12927).^71–74^ The Medusa/Spana software package was applied for calculation of Pourbaix (pe-pH_m_) diagrams.^75,76^

Density functional theory (DFT) using the B3LYP functional^77,78^ was employed to probe 1 : 1 and 1 : 2 complexes between Pu(iii) and EDTA as a function of pH and calcium concentration. Plutonium was described by an f-in-core pseudo potential ECP83MWB with corresponding basis sets of triple-zeta quality.^79,80^ All other atoms were described by the def2-TZVP basis sets as implemented in the TURBOMOLE software package.^81^ After the structural optimization, all geometries were proven to be true minima by vibrational frequency calculations.

## 4. Results and discussion

### 4.1 Experiments conducted in the absence of calcium

Curium fluorescence spectroscopy and Pu(OH)_3_(am) solubility studies were conducted in the presence of EDTA to define the aqueous chemistry of the Cm(iii)– and Pu(iii)–EDTA systems. These studies targeted alkaline conditions to determine the impact of hydrolysis on Cm(iii)– and Pu(iii)–EDTA complexes in the absence of calcium.

#### 4.1.1 Curium TRLFS as a function of pH in the presence of EDTA

**Fig. 1:**
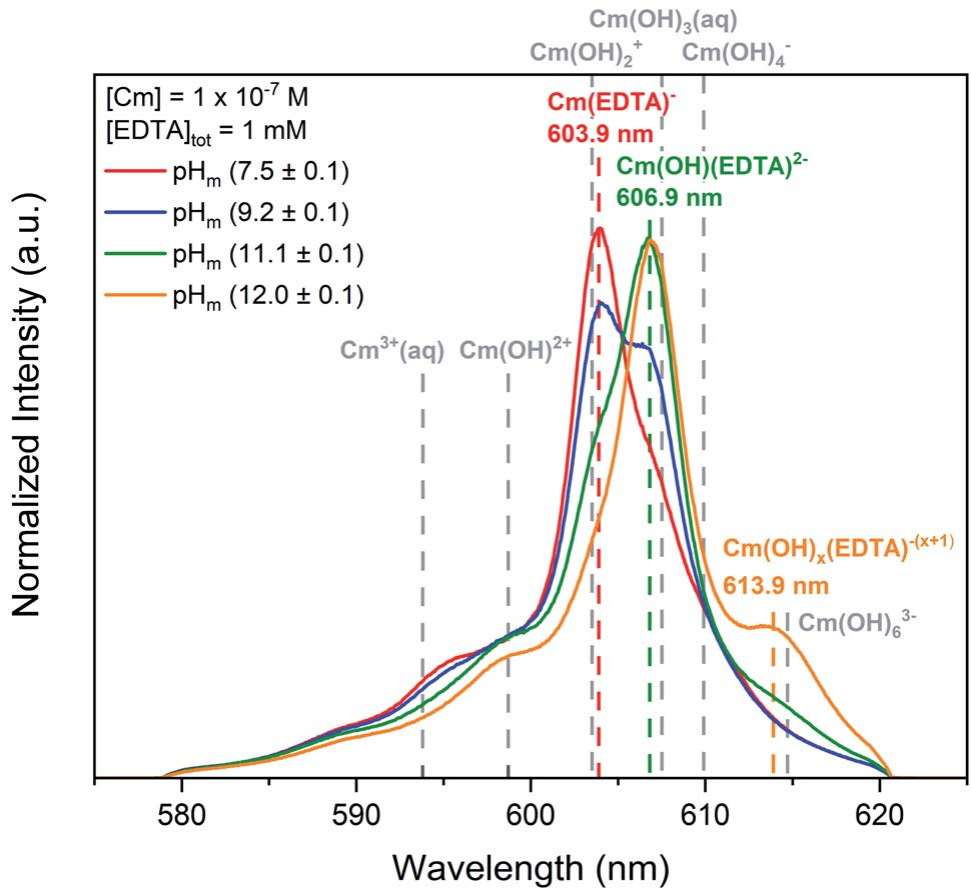
Laser fluorescence spectra of curium ([Cm(iii)]_tot_ = 10^−7^ M) equilibrated with [EDTA]_tot_ = 1 mM at *I* = 0.1 M as a function of pH_m_ (7.5–12.0). Cm–EDTA peak positions are identified by colored dashed lines and peak positions for curium hydrolysis species (dashed grey lines) are provided for comparison.^52,68,82,83^

Fig. 1 shows time-resolved laser fluorescence spectroscopy (TRLFS) spectra of experiments containing 10^−7^ M curium equilibrated with solutions of 1 mM EDTA at 7.5 ≤ pH_m_ ≤ 12.0. A comparison of the TRLFS peak positions, species identifications, lifetimes, and number of coordinated water molecules for curium hydrolysis and EDTA species is provided in Table 1.

**Table 1:** Summary of TRLFS peak positions, species assignments, lifetimes, and numbers of coordinated water molecules for the curium aqua ion, curium hydrolysis products, and curium–EDTA species

Species	Peak maxima position (nm)	pH	[Ca(ii)]_tot_ (used for eval.)	Lifetime (μs)	*N*_H_2_O_ (±2*σ*)
Cm^3+^	593.8^d^^,^^e^	≤8	—	64 ± 3^e^	9.1^f^
Cm(OH)^2+^	598.7^d^^,^^e^	8–9	—	76 ± 2^e^	—
Cm(OH)_2_^+^	603.5^d^	8–12	—	80 ± 20^e^	—
Cm(OH)_3_(aq)	607.5^e^	9–12	—	—	—
Cm(OH)_4_^−^	609.9^e^^,^^g^	10–12.5	—	—	—
Cm(OH)_6_^3−^	614.7^e^^,^^g^	≥11	—	—	—
Cm(EDTA)^−^	603.9^a^^,^^c^	≤7	0 M	231 ± 40^a^^,^^c^	2.1 ± 0.7^a^^,^^c^
		3.8–8.0	—	138 ± 5^c^	3.8^c^
Cm(OH)(EDTA)^2−^	606.9^a^^,^^c^	9–12	0 M	332 ± 41^a^^,^^c^	1.1 ± 0.3^a^^,^^c^
		9.0	—	212 ± 5^c^	2.2^c^
Cm(OH)_*x*_(EDTA)^−(*x*+1)^	613.9^a^^,^^c^	≥12	0 M	173^a^^,^^b^^,^^c^	2.9^a^^,^^b^^,^^c^
Ca–Cm–EDTA	603.8^a^	7–11	≥1 mM	150 ± 11^a^	3.5 ± 0.3^a^
Ca–Cm–OH–EDTA	610.1^a^	≥12	≥10 mM	813 ± 280^a^	0.1 ± 0.1^a^

a Observed in this work.

b Evaluated upon the pH = 12 spectra only, hence, related uncertainty is expected to be rather large.

c Lifetime and DFT calculations discussed in Trumm *et al.*^84^

d Fanghänel *et al.*^82^

e Rabung *et al.*^83^

f Thakur *et al.*^52^

g Described in Neck *et al.*^68^ as Ca_2_[Cm(OH)_4_]^3+^ and Ca_3_[Cm(OH)_6_]^3+^.

At pH_m_ = (7.5 ± 0.1), the curium species expected in the absence of EDTA is Cm^3+^, which has a fluorescence peak at 593.8 nm and a lifetime of 64 μs.^82,83^ While a minor peak in this general region of the TRLFS spectrum is observed, the majority of the signal was centered at 603.9 nm with a lifetime of (231 ± 40) μs. The ∼10 nm shift to higher wavelength and elongated lifetime signifies that a Cm–EDTA species, likely Cm(EDTA)^−^, has formed. This species was calculated to have 2.1 coordinated water molecules. Thakur *et al.*^52^ reports a shorter lifetime (138 ± 5 μs) and a larger number of coordinated water molecules (*N*_H_2_O_ = 3.8) for the Cm(EDTA)^−^ species, but investigated the Cm–EDTA system at lower pH (pH = 3.6), higher Cm(iii) concentrations (4 × 10^−6^ M), and much lower EDTA concentrations (1.6 × 10^−5^ M) than the present study. Thus, a direct comparison of the data beyond the reported lifetimes and wavelengths is not feasible.

Additional spectral changes were observed with increasing pH_m_. The prominent peak at 603.9 nm disappeared and was replaced by another peak at 606.9 nm, resulting in an isosbestic point at ∼605 nm. The existence of this isosbestic point indicates the presence of two distinct species in equilibrium: Cm(EDTA)^−^ predominated at pH_m_ <(9.2 ± 0.1) and fluoresced at 603.9 nm, and a second species gained predominance at pH_m_ >(9.2 ± 0.1) and fluoresced at 606.9 nm. As the only parameter varied across the series of samples was the pH_m_, it is logical to propose that the equilibrium between the two identified species must involve the coordination of an OH^−^ ion, indicating that the formation of a Cm–OH–EDTA ternary species took place in the system. However, we must also consider that this isosbestic point may instead represent an equilibrium between the 1 : 1 Cm(EDTA)^−^ and a 1 : 2 Cm(EDTA)_2_^5−^ complex. At elevated pH, the free concentration of the fully deprotonated ligand, [EDTA^4−^]_free_, increases due to the fourth deprotonation constant of H_4_EDTA (*i.e.*, fraction of fully deprotonated ligand ≤37% at pH ≤9.2 and 37–100% EDTA^4−^ at pH ≥9.2), and this increase could in turn lead to the chelation of two EDTA ligands to the metal center of the complex. M^III^(EDTA)_2_^5−^ complexes have been proposed previously in the literature for Pu(iii) and Am(iii),^32,57^ and are thus within the realm of possibility within this study.

The lifetime of the species corresponding to the 606.9 nm peak was determined to be (332 ± 41) μs, which translates to 1.1 coordinated water molecules present in the first shell of the complex. The decrease in the number of water molecules compared to Cm(EDTA)^−^ (from 2.1 to 1.1) provides indirect evidence for the replacement of a coordinated water molecule with an OH^−^ ion. In our recent study, the calculated vibronic side bands of both the hydroxo-species and the 1 : 2 complex shared similar features with the measured signal; however, the overall conclusion in combination with DFT calculations suggested that stepwise hydrolysis of Cm–EDTA complexes occurs with increasing alkalinity.^84^ If we instead assume the formation of the 1 : 2 complex, the coordination environment of Cm(iii) would be saturated due to the chelation of two EDTA ligands, which is expected to significantly increase the fluorescence lifetime of the complex. This would in turn, result in a calculated number of coordinated water molecules close or equal to 0, in disagreement with our experimental observations. The experimental findings herein are also in line with those of the Cm(iii)–TRLFS study published by Thakur and co-workers, who reported the predominance of the species Cm(OH)(EDTA)^2−^ at pH = 9 in 0.1 M NaClO_4_.^52^ The ternary complex M(OH)(EDTA)^2−^ has been also proposed in the literature for trivalent europium and americium,^40,44,56,57^ and is consistent with the stepwise hydrolysis of metal–EDTA species under alkaline pH.^9,85–90^ Therefore, it is our hypothesis that the complex observed to fluoresce at 606.9 nm within this study is the Cm(OH)(EDTA)^2−^ species, which forms *via* the coordination of a single OH^−^ ion to the binary complex. However, we do acknowledge the necessity of a more detailed investigation on the system as a function of the EDTA total concentration.

A third, relatively weak peak was observed at 613.9 nm for experiments at pH_m_ = (12.0 ± 0.1). The presence of this additional feature suggests another stepwise hydrolysis species, Cm(OH)_*x*_(EDTA)^−(*x*+1)^. The lifetime of this species was smaller than those of the two previously mentioned Cm(–OH)–EDTA species, thus supporting the hypothesis that an additional Cm–OH–EDTA species may form under hyperalkaline conditions. However, the lifetime and number of coordinated water molecules were calculated from only a single spectral analysis at pH_m_ (12.0 ± 0.1), resulting in large associated uncertainties. From these observations, we assign this species as Cm(OH)_*x*_(EDTA)^−(*x*+1)^, following the previously observed trend of hydrolysis. Ultimately, Cm–EDTA TRLFS spectra provided evidence for the formation of multiple potential Cm(–OH)–EDTA species in solution at pH_m_ = 7.5–12.0: Cm(EDTA)^−^, Cm(OH)(EDTA)^2−^, and Cm(OH)_*x*_(EDTA)^−(*x*+1)^.

#### 4.1.2 Pu(OH)_3_(am) pe-pH_m_ measurements

Experimentally-determined pH_m_ and pe values from Pu(OH)_3_(am) solubility experiments are plotted on the Pourbaix diagram in Fig. S1,† which shows the calculated predominance fields of plutonium solid compounds and aqueous species with a total EDTA concentration ([EDTA]_tot_) of 1 mM and 0 M CaCl_2_. The experimental pH_m_, initially adjusted to pH_m_ = 9, shifted to lower pH over time, resulting in equilibrium pH_m_ values of ∼7.6 for solutions containing dithionite (DT) and ∼7.3 for experiments containing Sn(ii). Measured *E*_h_ values ranged from −0.34 to −0.10 (±0.05) V for solutions containing both DT and Sn(ii) over the lifetime of the experiments. The Pourbaix diagram predicts the predominance of aqueous Pu^III^(EDTA)^−^ under our experimental conditions.

The NEA-TDB does not select a Pu(iii)–OH–EDTA complex, and to date, none have been experimentally identified within the literature. The only discussion of a Pu(iii)–OH–EDTA complex is described by Hummel,^53^ where the author performed a series of non-linear free energy relationship analyses with literature values for other lanthanides and actinides to postulate the formation of this complex and propose an estimated formation constant. The experimental Cm(iii)–EDTA TRLFS results discussed above suggest that hydrolyzed Pu(iii)–EDTA complexes could form at pH_m_ ≥9. The addition of hydrolyzed Pu(iii)–OH–EDTA complexes to the calculations for the Pourbaix diagram is expected to shift the Pu(iii)/Pu(iv) redox boundary to slightly more oxidizing conditions at alkaline pH, affecting the plutonium redox distribution under repository-relevant conditions. As the corresponding formation constants of these Pu(iii)–OH–EDTA species are lacking, the given complexes cannot be displayed in Fig. S1.†

#### 4.1.3 Pu(OH)_3_(am) aqueous-phase characterization by X-ray absorption spectroscopy

Fig. S2† shows the Pu L_III_-edge X-ray absorption near edge structure (XANES) spectrum for the aqueous phase from a Pu(OH)_3_(am) solubility experiment with 1 mM EDTA and 0 M CaCl_2_ at pH_m_ ≈ 7.6 together with reference spectra of Pu(iii)_aq_ and Pu(iv)_aq_.^66^ The comparison of the XANES spectra collected for the aqueous phase of the solubility experiment to the reference spectra shows that the experimental solution contained a combination of both the +iii and +iv oxidation states of plutonium. The energy of the sample white line (E_WL_) exhibited a 2 eV shift towards higher photon energy as compared to the Pu(iii)_aq_ reference spectrum, which is larger than the typical energy calibration error (±0.5 eV) (Table S5†), as did the position of the first oscillation with respect to *E*_WL_ (∼1.5 eV shift). This indicates that partial oxidation to Pu(iv) occurred in solution.

Oxidation of Pu(iii) to Pu(iv) in the presence of EDTA has been reported in the literature and proposed to be driven by the generation of radiolytically-produced, oxidizing byproducts of water and/or the preferential stability of Pu(iv)–EDTA complexes.^10,11,91^ Since the literature studies that noted radiolysis-based effects were primarily conducted with ^239^Pu (*t*_1/2_ = 2.41 × 10^4^ a), we chose to use the less-active isotope ^242^Pu (*t*_1/2_ = 3.75 × 10^5^ a) within our experiments to allow for the exclusion of a majority of these potential radiolytic effects.^92^ Unlike in previous studies, the aqueous redox conditions within the present study were controlled using two different redox buffers—DT and Sn(ii). In the very reducing conditions defined by both redox buffers, thermodynamic calculations reflected within the Pourbaix diagram (Fig. S1†) predicted only the predominance of aqueous Pu(iii)–EDTA species. The experimentally observed partial oxidation remains unexplained, although two hypotheses are raised: (i) potential intra-complex redox reaction involving the chemical oxidation of plutonium and reduction of EDTA, similar to the intra-complex redox reactions proposed in our previous work,^11^ and/or (ii) minor formation of Pu(iv) colloids or particulates which act as nucleation points for the PuO_2_(am,hyd) solid phase that is calculated to be thermodynamically stable under these conditions.

#### 4.1.4 Undersaturation Pu(OH)_3_(am) solubility experiments in the presence of EDTA

**Fig. 2:**
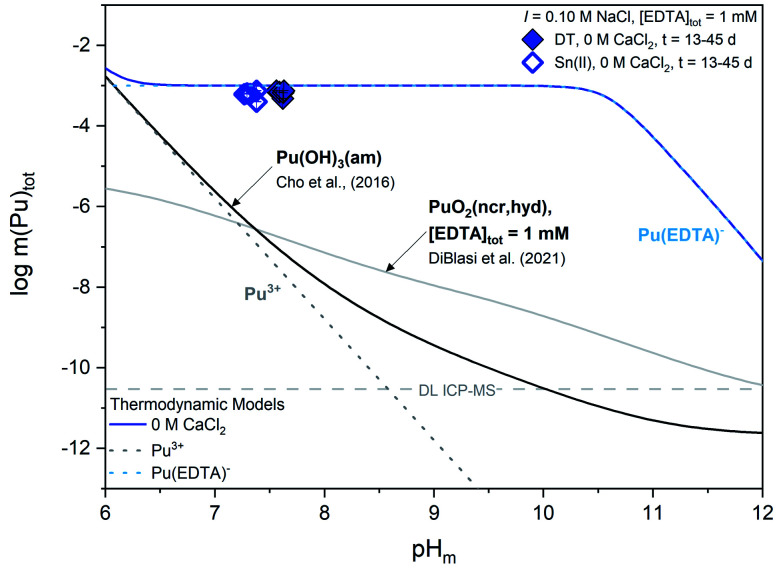
Experimentally measured *m*(Pu)_tot_ for Pu(OH)_3_(am) equilibrated with a solution containing [EDTA]_tot_ = 1 mM, *I* = 0.1 M NaCl, and 0 M CaCl_2_. Solid data symbols represent experiments containing dithionite (DT) as the redox buffer while open data symbols represent experiments containing Sn(ii) as the redox buffer. Solid lines correspond to the calculated solubility line of plutonium under different solution conditions and dotted lines correspond to the predicted individual concentrations of the various plutonium-containing solution species. Gray dashed lines represent the ICP-MS detection limits for the experimental analyses and all error bars are contained within data symbols.

Fig. 2 shows aqueous-phase total molal concentrations of plutonium (*m*(Pu)_tot_) in equilibrium with Pu(OH)_3_(am) in solutions containing 1 mM EDTA, 0 M CaCl_2_, and redox buffer (DT or Sn(ii)). The calculated solubility of Pu(OH)_3_(am) and PuO_2_(ncr,hyd) in the presence of 1 mM EDTA, which correspond to Pu(iii)–EDTA and Pu(iv)–EDTA species in solution, respectively, are provided for comparison.^12,14,21,93^ Steady state was achieved within 13 days.

Experimental *m*(Pu)_tot_ data are clearly above the calculated solubility of both Pu(OH)_3_(am) in the absence of EDTA and PuO_2_(ncr,hyd) in the presence of EDTA, indicating that the solution concentration of plutonium is governed by the formation of Pu(iii)–EDTA complex(es) through the dissolution of the Pu(OH)_3_(am) phase. This observation supports the expected formation of Pu^III^(EDTA)^−^ and is thus consistent with experimental (pe + pH) values and thermodynamic calculations. Based on the XANES results in Fig. S2,† we might expect total aqueous plutonium concentrations to fall between the calculated solubility curves for Pu(OH)_3_(am) and PuO_2_(ncr,hyd) in the presence of 1 mM EDTA instead of complete agreement between *m*(Pu)_tot_ and the calculated solubility of Pu(OH)_3_(am) with 1 mM EDTA. Additionally, thermodynamic calculations in Fig. S1† show the expected predominance of PuO_2_(am,hyd) in most of the (pe + pH) conditions investigated in this work. In a series of solubility experiments with PuO_2_(ncr,hyd), Sn(ii), and absence of EDTA, Tasi and co-workers reported the co-existence of Pu(iii) and Pu(iv) in the solid phase after a contact time of 146 days.^59^ The mismatches obtained between some of our observations (*i.e.*, measured solubility *vs.* oxidation state) and calculations (*i.e.*, predicted Pu(iv) solid phase) outlined within this paragraph may reflect the transient character of the Pu(iii) solubility data, which possibly evolves in the long-term towards a mixed Pu(iv)–Pu(iii) system. In this respect, the choice by Rai and co-workers of using PuPO_4_(cr) as the solid phase to investigate the Pu(iii)–EDTA system seems appropriate due to the increased stability field of the Pu^III^PO_4_(cr) ⇔ Pu(iii)–EDTA(aq) equilibrium, at least up to pH ≈ 10–11 (Fig. S3†).

#### 4.1.5 Thermodynamic modeling

The identification of M(iii)–OH–EDTA complex(es) has been previously reported for many relevant systems, such as europium, americium, and curium,^40,44,52,54,56–58^ and is complimentary to the reported hydrolysis of Pu(iv)–EDTA complexes at alkaline pH.^9,12,90^ Building upon the previously reported Cm(iii)–EDTA speciation, the TRLFS results described above enabled us to derive an approximate value for the equilibrium constant of the Cm–OH–EDTA complex. The evaluation was carried out through spectral deconvolution of the sum area normalized spectra involving Lorentzian peak fitting with fixed peak locations and full-width at half maxima (FWHM) for the signals located at 603.9, 606.9, and 613.9 nm as assigned to the Cm(EDTA)^−^, Cm(OH)(EDTA)^2−^, and Cm(OH)_2_(EDTA)^3−^ complexes, respectively. The deconvoluted peak areas were summed and correlated with the total curium concentration in solution to calculate the individual concentrations and fractions of the complex species. This procedure was conducted under the assumptions that only three species (*i.e.*, Cm(EDTA)^−^, Cm(OH)(EDTA)^2−^, and Cm(OH)_2_(EDTA)^3−^) form under the given conditions, and the sum area normalization of the spectra corrected for any minor differences in fluorescence intensity factors that were identified between the species. As observed experimentally, we expect an equal distribution of the Cm(EDTA)^−^ and Cm(OH)(EDTA)^2−^ species at pH ≈ 9.5. The formation constant generated through spectral deconvolution resulted in an equal distribution point between these two species at pH ≈ 9.6 (Fig. S4†). This agreement between the calculated and observed equal distribution point provided further confirmation that the speciation distribution was correctly reproduced by the procedure with the applied assumptions. The equilibrium constant corresponding to the step-wise hydrolysis reaction of Cm(EDTA)^−^ (eqn (3)) was determined at each investigated pH using the ionic strength corrected (SIT) activities of the species and averaged to produce the final value of log *K*°(Cm(OH)(EDTA)^2−^) = (4.0 ± 1.9).

3 Cm(EDTA)^−^ + OH^−^ ⇔ Cm(OH)(EDTA)^2−^, log *K*_Cm_° = (4.0 ± 1.9)

While the equilibrium constant for the hydrolysis of the Cm(EDTA)^−^ complex at alkaline pH was determined with relatively large uncertainty (2σ, 95% confidence), a qualitative comparison to the literature shows that the mean value is comparable to other analogous values, such as the log *K*_Eu_° (eqn (4)) and log *K*_Am_° (eqn (5)) values reported for the hydrolysis of Eu(EDTA)^−^ and Am(EDTA)^−^, respectively.^54,58^ Additionally, Hummel^53^ proposed estimated log *K*° values for Cm(iii) and Pu(iii) (eqn (6) and (7)) from non-linear free energy relationship analyses; these values also align reasonably well with the calculated equilibrium constant resulting from the Cm(iii) TRLFS study.

4 Eu(EDTA)^−^ + OH^−^ ⇔ Eu(OH)(EDTA)^2−^, log *K*_Eu_° = 4.87

5 Am(EDTA)^−^ + OH^−^ ⇔ Am(OH)(EDTA)^2−^, log *K*_Am_° = (2.62 ± 0.13)

6 Cm(EDTA)^−^ + OH^−^ ⇔ Cm(OH)(EDTA)^2−^, log *K*_Cm_° <3

7 Pu(EDTA)^−^ + OH^−^ ⇔ Pu(OH)(EDTA)^2−^, log *K*_Pu_° <4

Pu(iii) solubility data collected in this work in the presence of EDTA are limited to a pH-region where no ternary Pu(iii)–OH–EDTA complexes are expected. For this reason, the validity of the speciation scheme proposed for the Pu(iii)–EDTA system was evaluated using the comprehensive dataset of Rai *et al.*^37^ (experimental conditions outlined in Section 2). The aim was to investigate the potential agreement between the data of Rai *et al.* and a new model including the formation of both the Pu(EDTA)^−^ complex, as defined by the authors, and a Pu(iii)–OH–EDTA species analogous to the one identified in the Cm(iii)–EDTA system.

Four different species were considered in the reevaluation of the solubility data from Rai *et al.*:^37^ Pu(HEDTA)(aq), Pu(EDTA)^−^, Pu(OH)(EDTA)^2−^, and Pu(OH)_2_(EDTA)^3−^. The fitting procedure determined that the inclusion of Pu(HEDTA)(aq) was not necessary to improve the description of the data set, and that the best fit was achieved by assuming only the formation of the Pu(EDTA)^−^ and Pu(OH)(EDTA)^2−^ species within the model. The data from Rai *et al.* was further evaluated as follows:

(i) optimization of log *β*°(Pu(EDTA)^−^) and log *β°(Pu(OH)(EDTA)^2−^) considering all the data reported by Rai *et al.*,

(ii) optimization of log *β*°(Pu(EDTA)^−^) and log *β°(Pu(OH)(EDTA)^2−^) with the data of Rai *et al.* excluding data at pH >12 and a single outlier at pH ∼4 (exclusion reasoning discussed below), and

(iii) comparison of the Rai *et al.* data with a model constructed by applying log *β*°(Pu(EDTA)^−^) from the NEA-TDB^21^ and log *β°(Pu(OH)(EDTA)^2−^) as derived in the present work from the Cm–EDTA TRLFS study with associated uncertainties.

Results of the three different modeling attempts are summarized in Table 2 along with a comparison of log *β*° values reported in the literature and derived in this work from Cm–EDTA TRLFS data. The log *β°_(1,1,1)_ value for model (iii) was calculated using the Cm–EDTA formation constant for the first hydrolysis product (eqn (3)) and the NEA-TDB log *β*°_(1,0,1)_ value for the unprotonated and unhydrolyzed Pu(iii)–EDTA complex, Pu(EDTA)^−^.^21^ The quality parameter represents the averaged square root of the sum of differences between the experimental and calculated plutonium concentrations, calculated as [∑(log[Pu]_exp_ − log[Pu]_calc_)^2^]^1/2^ × (*n* − 1)^−1^, where *n* is the number of data points.

**Table 2:** Modeling results (log *β°) and statistical measures of the fit for Pu(iii)–EDTA solubility from PuPO_4_(cr,hyd) as compared to log *β° reported in the literature or derived within this work from Cm–EDTA TRLFS data

Source	Model reactions	log *β*°	*R* ^2^	Quality parameter^c^
NEA-TDB	Pu^3+^ + EDTA^4−^ + H^+^ ⇔ Pu(HEDTA)(aq), Pu^3+^ + EDTA^4−^ ⇔ Pu(EDTA)^−^	(22.02 ± 0.45)^a^, (20.18 ± 0.37)^a^	0.7658	0.0449
Rai *et al.*^37^	Pu^3+^ + EDTA^4−^ ⇔ Pu(EDTA)^−^	(19.97 ± 0.62)	0.7685	0.0444
Model (i): all data	Pu^3+^ + EDTA^4−^ ⇔ Pu(EDTA)^−^	(20.62 ± 0.08)^b^	0.8502	0.0240
	Pu^3+^ + EDTA^4−^ + H_2_O(l) ⇔ Pu(OH)(EDTA)^2−^ + H^+^	(6.36 ± 1.13)^b^		
Model (ii): data pH <12	Pu^3+^ + EDTA^4−^ ⇔ Pu(EDTA)^−^	(20.47 ± 0.05)^b^	0.8727	0.0136
	Pu^3+^ + EDTA^4−^ + H_2_O(l) ⇔ Pu(OH)(EDTA)^2−^ + H^+^	(9.02 ± 0.13)^b^		
Model (iii): data from NEA-TDB^21^ and Cm(iii)–EDTA TRLFS	Pu^3+^ + EDTA^4−^ ⇔ Pu(EDTA)^−^	(20.18 ± 0.37)^a^	0.5096	0.0426
	Pu^3+^ + EDTA^4−^ + H_2_O(l) ⇔ Pu(OH)(EDTA)^2−^ + H^+^	(10.2 ± 2.0)^b^		

a Ref. 21

b Derived in the current work.

c Quality parameter = [∑(log[Pu]_exp_ − log[Pu]_calc_)^2^]^1/2^ × (*n* − 1)^−1^.

**Fig. 3:**
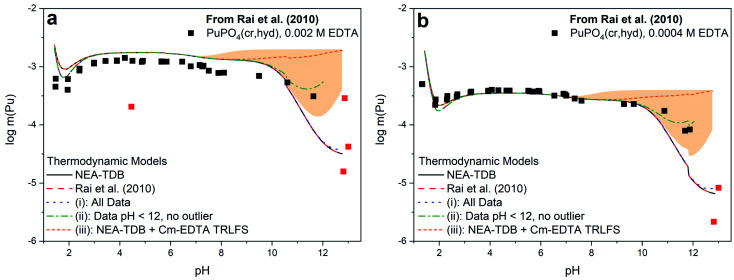
PuPO_4_(cr,hyd) solubility in equilibrium with (a) 0.002 M EDTA or (b) 0.0004 M EDTA from Rai *et al.*^37^ as compared to the calculated solubility using thermodynamic models from the NEA-TDB,^21^ the original publication,^37^ or derived in this work from modeling exercises (i)–(iii). The orange shaded region represents the uncertainty associated with the formation constant generated from Cm(iii)–EDTA TRLFS data and data points excluded from model (ii) are displayed in red.

Fig. 3 shows a comparison of the different models summarized in Table 2 with the solubility data of Rai *et al.*^37^ Models constructed from (i) and (ii), which include the first hydrolysis complex, exhibited slightly improved *R*^2^ and quality parameter values compared to those constructed from literature and Cm–TRLFS values. The best description of the data set was achieved when hyperalkaline data and the outlier data point at pH ∼4 were excluded from the fitting (*i.e.*, model (ii)). Even so, the log *β°_(1,1,1)_ value for this model predicts the predominance of Pu(OH)(EDTA)^2−^ only at pH >11, indicating that this formation constant was calculated from only 3 data points and thus, has limited reliability. The model constructed from Cm–EDTA TRLFS studies and NEA-TDB values (*i.e.*, model (iii)) had the least favorable fitting statistics. However, the log *β°_(1,1,1)_ derived from the Cm–EDTA TRLFS study had such large associated uncertainty that, while the model significantly overpredicted the solubility data at pH >9, the uncertainty (shaded area in Fig. 3) covered a wide enough range that it ultimately included the data points at pH 11–12. Therefore, the models constructed from (i)–(iii) can neither confirm nor deny the existence of a Pu(iii)–OH–EDTA complex.

The discrepancy between the generated models, which describe a system where Pu(iii)–OH–EDTA complexes only become predominant above pH 11, and the Cm–EDTA TRLFS study, which displayed clear evidence of hydrolysis occurring at a pH as low as 9, is puzzling. Due to this disagreement, we argue that the hyperalkaline data of Rai *et al.*^37^ may not be sufficient for fitting the hydrolysis species Pu(OH)(EDTA)^2−^ because data points at pH >12 are limited and total plutonium concentrations vary by up to 1.0 log_10_ units at nearly identical pH values. It was for this reason that model (ii) was constructed excluding data points at pH >12. We believe it is possible that Rai *et al.* encountered oxidation state changes that may have significantly affected their hyperalkaline data points. During their experiments, Rai *et al.* utilized both HQ and DT in solution as a reducing agents and extensively characterized their aqueous plutonium oxidation state distribution using traditional solvent extraction techniques coupled with KBrO_4_ to quantify the fraction of Pu(iii) in solution. The authors reported 1.9–8.1% Pu(iv) in solution for samples with pH ≥9 (with one sample having 41.5% Pu(iv)), indicating that oxidation from Pu(iii) to Pu(iv) at high pH may have occurred. While this Pu(iv) fraction initially appears to be negligible, it is possible that the presence of Pu(iv) under high pH conditions may have significantly impacted the observed solubility data. Pu(iii)–EDTA complexes tend to be more soluble than Pu(iv)–EDTA complexes under most pH conditions, but this discrepancy is more pronounced at alkaline pH (*e.g.*, Pu(iii)–EDTA ≈ 10^−4^ m *vs.* Pu(iv)–EDTA ≈ 10^−9^ m for 1 mM EDTA solutions at pH 11).^9,12,37,90^ We believe that the oxidation to Pu(iv) observed by Rai *et al.* may have resulted in oversaturation and subsequent precipitation of PuO_2_(am,hyd), thus significantly decreasing the experimental solubility under hyperalkaline conditions. The possible transformation of PuPO_4_(cr) into PuO_2_(am,hyd) above pH ≈ 11 is also implied in the Pourbaix diagram calculated for the Pu–PO_4_–EDTA system (Fig. S3†), which includes the (pe + pH) values reported by Rai *et al.*^37^

In this context, additional solubility data with well-described plutonium oxidation state distributions are necessary in the hyperalkaline region to properly assess the formation, stoichiometry, and stability constants of Pu(iii)–OH-EDTA species. As described in both this work and in the literature, maintaining constant plutonium oxidation state under hyperalkaline conditions is non-trivial.^25,37^ Since we are lacking this reliable data, a linear free energy relationship (LFER) comparison (Fig. S5†) was constructed to evaluate the stability constants of Pu(iii)–OH–EDTA complex(es) generated within this work; the formation constant from model (ii) was closest to the LFER predicted value. This comparison to literature values for other trivalent actinides and lanthanides,^37,54,58^ while limited in nature, not only provides additional qualitative support that these complex(es) form, but also helps isolate an expected formation constant value for the species where experimental data was insufficient. For these reasons, continued investigation with Pu(iii) analogs, such as Cm(iii) or Am(iii), coupled with the continued use of LFER with reliable experimental datasets, are expected to provide further insight into the speciation and solubility of An(iii)–EDTA systems in alkaline to hyperalkaline systems.

### 4.2 Experiments conducted as a function of calcium concentration

Cm–EDTA TRLFS and Pu(OH)_3_(am)–EDTA solubility studies were conducted in the presence of calcium to probe the impact of Ca(ii) on An(iii)–EDTA systems (An = Cm or Pu). While the literature generally assumes that Ca(ii) will compete with curium and plutonium for EDTA complexation, our previous study indicated that calcium stabilizes binary An(iv)–EDTA complexes in solution.^12^ This, coupled with additional evidence within the literature regarding calcium-stabilized ternary M–L complexes,^22–27^ suggests the possibility of ternary Ca–An(iii)–EDTA and/or quaternary Ca–An(iii)–OH–EDTA complex formation under the conditions investigated within this study.

#### 4.2.1 Curium TRLFS as a function of pH in the presence of EDTA and calcium

**Fig. 4:**
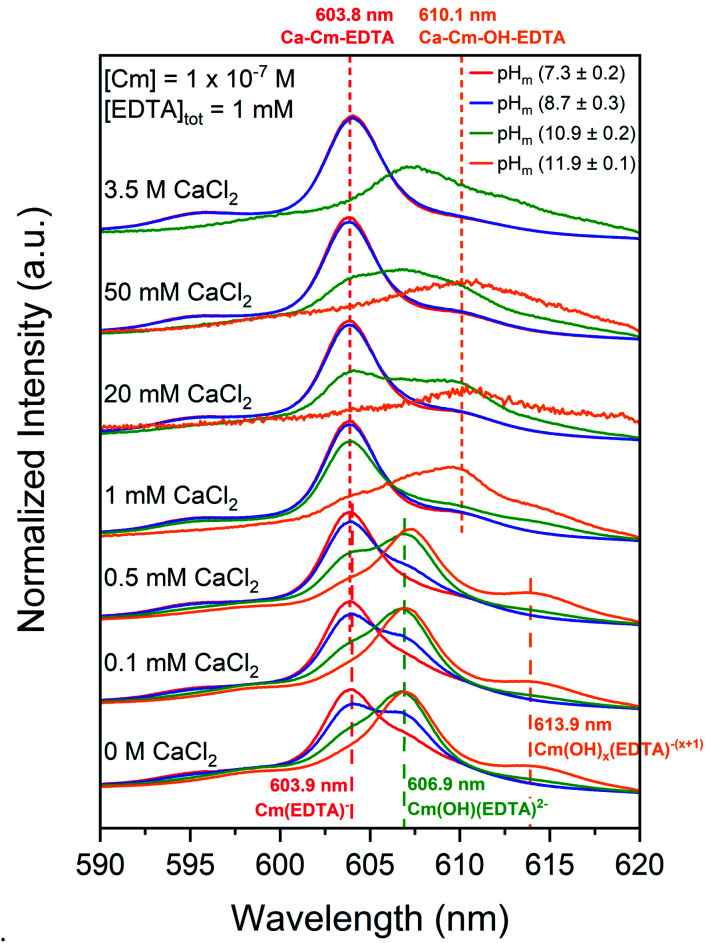
Laser fluorescence spectra of curium ([Cm(iii)]_tot_ = 10^−7^ M) equilibrated in solutions containing [EDTA]_tot_ = 1 mM and *I* = 0.1 or 10.5 M at pH_m_ 7.3, 8.7, 10.9, or 11.9 as a function of calcium concentration (0 M ≤ [CaCl_2_] ≤ 3.5 M). Cm–EDTA peak positions are identified by dashed vertical lines.

Fig. 4 shows TRLFS spectra of experiments containing [Cm(iii)]_tot_ = 10^−7^ M in equilibrium with solutions containing 1 mM EDTA as a function of pH_m_ (7.3, 8.7, 10.9, or 11.9) and calcium concentration (0 M ≤ [CaCl_2_] ≤ 3.5 M). The fluorescence peaks for aqueous Cm–EDTA species (from Fig. 1) are shown for comparison.

When calcium concentration was increased from 0 M to 1 mM CaCl_2_, the intensity of the previously discussed Cm–OH–EDTA peak (606.9 nm, Section 4.1.1) decreased and was replaced by a peak of similar wavelength position to the Cm(EDTA)^−^ peak (603.8 nm). The 606.9 nm hydrolysis complex peak location and fluorescence lifetime remained unaffected by the addition of calcium, indicating that the changes in TRLFS spectra as a function of calcium concentration likely represent an equilibrium between the Cm–OH–EDTA species and a novel species at 603.8 nm. This peak at *λ* = 603.8 nm maintained predominance in systems with high total calcium concentrations; pH_m_ = (7.3 ± 0.2) and pH_m_ = (8.7 ± 0.3) experiments at [CaCl_2_] ≥1 mM show only the presence of the 603.8 nm peak. This trend is also clear for experiments at pH_m_ = (10.9 ± 0.2) with [CaCl_2_] ≤1 mM.

The calculated lifetime of the species that fluoresces at 603.8 nm in the presence of calcium (150 ± 11 μs) is shorter than for Cm(EDTA)^−^ (231 ± 40 μs). This indicates differences in the coordination environment between the two complexes since the coordination environment of the first shell of the complex is the primary factor in determining the fluorescence signal relaxation time. These results suggest that a Ca–Cm–EDTA complex forms in solution under the investigated experimental conditions. When comparing the number of coordinated water molecules, the species in the presence of calcium is calculated to have *N*_H_2_O_ = 3.5, the Cm(EDTA)^−^ species without calcium is calculated to have *N*_H_2_O_ = 2.1, and the Cm(OH)(EDTA)^2−^ species without calcium is calculated to have *N*_H_2_O_ = 1.1. The different coordination environments for Cm(iii) in the unhydrolyzed and hydrolyzed Cm–EDTA complexes further proves that the signal observed at 603.8 nm with increasing total calcium concentration is indeed a different solution species. Since we cannot neglect the possibility that calcium may stabilize either Cm(EDTA)^−^ or Cm(OH)(EDTA)^2−^, and further investigation is necessary to define the stoichiometry of this calcium–containing complex, it is only labeled as a Ca–Cm–EDTA species within this work.

Fig. S6† shows a comparison of sum area normalized TRLFS spectra at each individual pH_m_ = (7.3–11.9) and the background corrected intensity spectra for pH_m_ = (10.9 ± 0.2) and (11.9 ± 0.1) analyses as a function of calcium concentration. The sum area normalized spectra at each pH_m_ (Fig. S6a–d†) highlight the formation of the Cm(OH)(EDTA)^2−^ (606.9 nm) and Cm(OH)_*x*_(EDTA)^−(*x*+1)^ (613.9 nm) complexes at low calcium concentrations and the Ca–Cm–EDTA complex (603.8 nm) at elevated calcium concentrations. In the pH_m_ = (11.9 ± 0.1) spectrum, the seemingly reversed equilibrium observed upon the addition of calcium resulted in a peak at 610.1 nm instead of the 603.8 nm peak shown in experiments with lower pH_m_. The peak at 610.1 nm was also observed in the pH_m_ = (10.9 ± 0.2) spectra, but under these conditions there was clear presence of both the 603.8 nm and 610.1 nm peaks, indicating a mixture of two different species. Considering the previously discussed tendency for metal–EDTA species to undergo hydrolysis, the additional peak at 610.1 nm is tentatively assigned to a quaternary Ca–Cm–OH–EDTA complex (Table 1).

We observed that the sum area normalized spectra for pH_m_ = (10.9 ± 0.2) and (11.9 ± 0.1) experiments at [Ca]_tot_ ≥1 mM lost spectral resolution (Fig. S6c and d†); this was due to the loss of overall intensity within these analyses, as represented by the background corrected intensities in Fig. S6e and f.† When calcium and EDTA were equimolar (*i.e.*, both 1 mM), aqueous curium concentrations decreased resulting in reduced fluorescence intensities. We expect that at high pH and calcium concentrations curium precipitated, most likely as a Ca–Cm–EDTA solid phase, thus explaining the decrease in fluorescence intensities observed under these conditions.

#### 4.2.2 Pu(OH)_3_(am) solid phase characterization

The solid phase from an experiment of Pu(OH)_3_(am) equilibrated in a pH_m_ = (9.0 ± 0.1) solution containing DT, 1 mM EDTA, and 20 mM CaCl_2_ was characterized by X-ray absorption near edge structure (XANES) spectroscopy (Fig. S7†). The white line energy (*E*_WL_) and first feature position (Table S6†) are in excellent agreement with the data reported for Pu^III^(OH)_3_(am), considering the typical energy calibration error (±0.5 eV), which shows that plutonium remained in the +iii oxidation state and no apparent solid transformation occurred under the experimental conditions of the solubility study within the time span of the investigation.

#### 4.2.3 Pu(OH)_3_(am) pe-pH_m_ measurements

Experimentally determined pH_m_ and pe values for Pu(OH)_3_(am) solubility studies containing both EDTA and calcium are plotted on the Pourbaix diagram in Fig. S8,† which shows the calculated predominance fields of plutonium solid compounds and aqueous species at different total calcium concentrations ([Ca]_tot_ = 1 mM or 20 mM) and 1 mM total EDTA concentration ([EDTA]_tot_). Shifts from pH_initial_ = 9 to lower pH_m_ values were observed, and this shift was more significant for DT-containing systems which was likely due to the disproportionation of DT (*i.e.*, 2S_2_O_4_^4−^ + H_2_O ↔ 2HSO_3_^−^ + S_2_O_3_^2−^).^94–97^ Measured *E*_h_ values ranged from −0.34 to −0.08 (±0.05) V for DT-containing experiments and −0.44 to −0.29 (±0.05) V for Sn(ii) containing experiments. WIPP simulated brine solutions equilibrated at pH_m_ ≈ 9.7 and *E*_h_ ≈ −0.16 V.

Experimental pe and pH_m_ measurements for 1 mM, 20 mM, and 3.5 M CaCl_2_ systems all fall within the Pu(iv) solid phase predominance field of PuO_2_(am,hyd) and the aqueous predominance field for Pu(iii)–EDTA species, indicating differences in predicted oxidation states between experimental solid and aqueous phases. WIPP simulated brine (pe + pH_m_) measurements fall within both the aqueous and solid phase Pu(iv) predominance region. Solid phase characterization, discussed in Section 4.2.2, suggests that the Pu(OH)_3_(am) solid phase used within these experiments did not exhibit any phase transformation over the lifetime of these experiments, and thus does not agree with the predicted plutonium oxidation state within the solid phase. As discussed for the calcium-free systems, the mismatch obtained between some of the experimental observations and thermodynamic calculations may reflect the transient character of the Pu(iii) solubility data, which may evolve in the long-term towards a Pu(iv)–Pu(iii) mixed system.

#### 4.2.4 Pu(OH)_3_(am) aqueous-phase characterization by X-ray absorption spectroscopy

The comparison of the XANES Pu L_III_-edge spectra collected for the aqueous phase of the Pu(OH)_3_(am) solubility experiment in equilibrium with 1 mM EDTA and 1 mM CaCl_2_ to the reference spectra for Pu(iii)_aq_ and Pu(iv)_aq_ (Fig. S9†) shows that the experimental solution contains only the +iii oxidation state of plutonium. The white line energy of the sample is consistent with the Pu(iii)_aq_ reference spectrum (Table S5†). These results differ from those observed for experiments conducted in the absence of calcium, where oxidation to Pu(iv) was observed in solution. In the Pu(iii)–EDTA system, calcium may stabilize Pu(iii)–EDTA complexes in solution and prevent oxidation to Pu(iv)–EDTA within the short time frames investigated within this study (≤45 days).

#### 4.2.5 Undersaturation Pu(OH)_3_(am) solubility experiments in the presence of EDTA and calcium

**Fig. 5:**
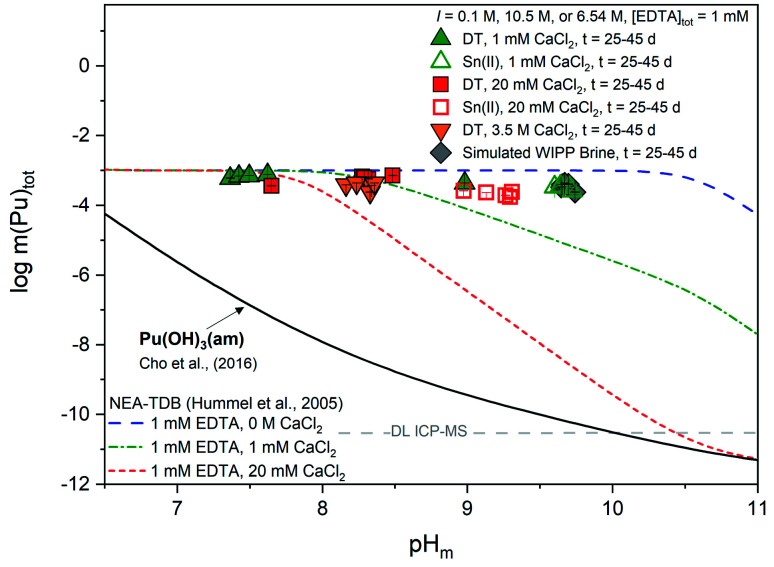
Experimentally measured *m*(Pu)_tot_ in equilibrium with Pu(OH)_3_(am) at [EDTA]_tot_ = 1 mM, *I* = 0.1 M NaCl (except 3.5 M CaCl_2_ and simulated WIPP brine) with 1 mM CaCl_2_ (green triangles), 20 mM CaCl_2_ (red squares), 3.5 M CaCl_2_ (orange triangles), or simulated WIPP brine (gray diamonds). Solid data symbols represent experiments containing dithionite (DT) while open data symbols represent experiments containing Sn(ii). Solid, dotted, and dashed lines correspond to the thermodynamically calculated solubility of Pu(OH)_3_(am) in the presence of EDTA with different calcium concentrations.^21,93^ Gray dashed lines represent the ICP-MS detection limits for the experimental analyses and all error bars are contained within data symbols.

Fig. 5 shows experimental concentrations of plutonium (*m*(Pu)_tot_) for Pu(OH)_3_(am) equilibrated in solutions containing 1 mM EDTA, redox buffer (DT or Sn(ii)), and calcium (1 mM ≤ [CaCl_2_] ≤ 3.5 M). The calculated solubility of both Pu(OH)_3_(am) in the absence of EDTA and Pu(OH)_3_(am) in the presence of 1 mM EDTA as a function of calcium concentration are provided for comparison. Steady state was achieved within 13 days.

**Fig. 6:**
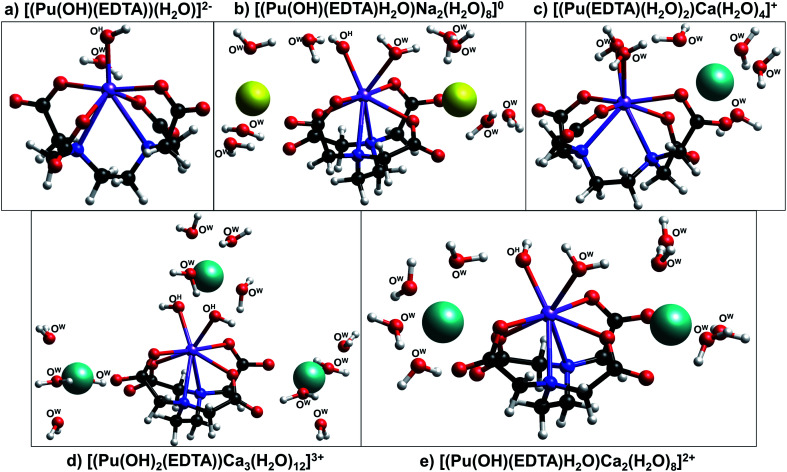
Ball-and-stick models of the DFT optimized structures (a) [Pu(OH)(EDTA)(H_2_O)]^2−^, (b) [Pu(OH)(EDTA)H_2_O)Na_2_(H_2_O)_8_]^0^, (c) [(Pu(EDTA)(H_2_O)_2_)Ca(H_2_O)_4_]^+^, (d) [(Pu(OH)_2_(EDTA))Ca_3_(H_2_O)_12_]^3+^, and (e) [(Pu(OH)(EDTA)H_2_O)Ca_2_(H_2_O)_8_]^2+^ calculated at the hybrid density functional theory level (B3LYP). Water and hydroxyl oxygen atoms are labeled with O^W^ and O^H^, respectively, while carboxyl oxygen atoms within the EDTA ligand are unlabeled. Legend: plutonium (purple), sodium (yellow), calcium (light blue), carbon (black), oxygen (red), nitrogen (dark blue), and hydrogen (white).

In the presence of EDTA and calcium, thermodynamic calculations using values from Tables S1 and S2† (*i.e.*, excluding the proposed ternary Ca–An(iii)–EDTA complex) predict a clear decrease in the solubility of plutonium with increasing calcium concentrations at pH_m_ ≥7.5 (green and red lines in Fig. 5). As discussed in Section 2, this is due to the formation of the Ca(EDTA)^2−^ complex, which effectively competes with plutonium for EDTA complexation and occupies all free EDTA^4−^ under these conditions. However, this trend is not reproduced by our experimental data. Instead, we observe a nearly pH_m_-independent trend limited by the inventory of the ligand in the system. This indicates that the presence of calcium does not, in fact, decrease the aqueous concentration of plutonium, but instead increases aqueous plutonium concentrations, likely through the formation of a calcium–containing complex. These observations provide additional indirect evidence on predominance of ternary Ca–Pu(iii)–EDTA and/or quaternary Ca–Pu(iii)–OH–EDTA complexes, and support the conclusions drawn from Cm(iii) TRLFS data.

Similar to our observations with the Pu(iv)–EDTA system,^12^ experiments conducted at high ionic strength (3.5 M CaCl_2_ and WIPP brine) exhibited higher plutonium concentrations than those predicted by thermodynamic calculations conducted for EDTA-free systems. Since these systems contained a large excess of calcium, and the Ca–EDTA complexes have high stability, these observations can only be explained by the formation of ternary Ca–Pu(iii)–EDTA and/or quaternary Ca–Pu(iii)–OH–EDTA complexes.

It is likely that the formation of calcium-containing Pu(iii)–EDTA complex(es) will affect the Pu(iii)/Pu(iv) redox borderline. As described in our previous work, the inclusion of quaternary Ca–Pu(iv)–OH–EDTA complex(es) within the model caused a significant shift in the previously-defined redox boundary for Pu(iii)/Pu(iv) towards lower (pe + pH_m_) values, thus increasing the stability field of Pu(iv)_aq_ species. Depending on the strength of the formation constants for possible Ca–Pu(iii)–EDTA complex(es), we would expect a similar shift towards more oxidizing values and an increase in the stability field of Pu(iii)_aq_ species. A larger predominance region for the more soluble Pu(iii)_aq_ species is of environmental and repository relevance, as it may significantly impact the fate and transport of plutonium. Further understanding the impact of this larger predominance region in more complex systems with repository relevant conditions and understanding how evolving redox environment, alkalinity, and organic concentrations from the near to the far field may affect this boundary shift are, therefore, of great importance for predicting the fate and transport of plutonium within these systems.

#### 4.2.6 Theoretical modeling

Finally, to further investigate the potential for calcium stabilization of Pu(iii)–EDTA complexes, density functional theory (DFT) was used to probe the structures of (Na)–Pu(iii)–OH–EDTA, Ca–Pu(iii)–EDTA, and Ca–Pu(iii)–OH–EDTA complexes at the hybrid density functional theory level (B3LYP). Five DFT-optimized structures are shown in Fig. 6. Pu(iii)–OH–EDTA structures in the absence of calcium were computed both without (Fig. 6a) and with (Fig. 6b) the presence of sodium to determine the role of sodium in aqueous complex stabilization and to further simulate the experimental conditions applied within this study (Section 4.1). These two complexes were structurally similar regardless of sodium inclusion, indicating that sodium ions may help stabilize, but likely will not change, Pu(iii)–(OH)–EDTA complexes in solution. EDTA chelated the plutonium center through metal–nitrogen and metal–oxygen bonds occupying 6 coordination sites, and additional water molecules or hydroxyl ions filled the rest of the inner coordination sphere of the complex. The primary difference between the complexes with and without sodium inclusion was the coordination number of the Pu(iii) center; in the absence of sodium, the plutonium was 7-coordinate, while in the presence of sodium, an additional water molecule coordinated to the plutonium center resulting in an 8-coordinate complex.

Three calcium-containing complexes optimized by DFT are illustrated in Fig. 6c–e. All three of these complexes were structurally similar to those without calcium and contain 8-coordinate plutonium centers, with 6 of the coordination sites being occupied by EDTA and the other two coordination sites occupied by either water or hydroxyl ions. It was observed that, as the number of hydroxyl ions associated to the metal center increased, the number of calcium ions required to stabilize the complex also increased; the complex containing no hydroxyl ions exhibited only one associated calcium ion, while the complex containing two hydroxyl ions required three calcium ions. Also, as more calcium ions were associated with the complex, plutonium–nitrogen bond lengths elongated, signifying a decrease in the strength of the bond (Table S7†). For both the sodium- and calcium-containing complexes, the counter ions tended to associate to the aqueous complex in two outer-sphere sites situated near the non-chelating carboxyl oxygens of EDTA; in the complex containing three calcium ions, the third calcium atom was situated above the two inner-sphere coordinated hydroxyl ions.

The combination of DFT, TRLFS, and undersaturation studies suggests the existence of four potential complex types within ternary Ca–An(iii)–EDTA systems: An(iii)–EDTA, An(iii)–OH–EDTA, Ca–An(iii)–EDTA, and Ca–An(iii)–OH–EDTA (An = Cm or Pu). While the exact stoichiometries and degree of ligand protonation within the proposed complexes remain experimentally undetermined, these DFT calculations provide additional qualitative support that both hydrolyzed and calcium-containing Pu(iii)–EDTA species can be chemically and structurally stable and highlight the complex nature of the system. Ultimately, this complexity underlines the importance of understanding ternary and quaternary interactions to accurately predict the fate and transport of radionuclides under environmentally- and repository-relevant conditions.

## 5. Conclusions

A combination of undersaturation solubility experiments with freshly prepared Pu(OH)_3_(am) solid phase and chemically controlled, well-defined redox conditions in solution, advanced spectroscopic and theoretical techniques, and a systematic study of Cm–EDTA fluorescence spectroscopy were considered to evaluate the chemical speciation of An(iii)–EDTA–H_2_O and Ca–An(iii)–EDTA–H_2_O systems (An = Cm or Pu). This evaluation suggests the formation of previously unreported An(iii)–OH–EDTA, Ca–An(iii)–EDTA, and Ca–An(iii)–OH–EDTA complexes.

The reference NEA-TDB project currently selects two Pu(iii)–EDTA complexes—Pu(EDTA)^−^ and Pu(HEDTA)(aq)—but M(iii)–OH–EDTA complexes have been previously proposed for different lanthanides and trivalent actinides (M = Eu, Am, and Cm). This work provides further fluorescence spectroscopic evidence that Cm(iii)–OH–EDTA complexes likely form at pH_m_ ≥9, suggesting the potential need for the inclusion of an analogous complex into the plutonium model. A reevaluation of literature solubility data determined that alkaline Pu(iii) solubility could be statistically described equally well with or without the inclusion of Pu(iii)–OH–EDTA species. The performed modeling exercise could not present unequivocal evidence on the formation of the hydrolyzed Pu(iii)–species. However, LFER using step-wise formation constants determined in this work for Cm(OH)(EDTA)^2−^ and available in the literature for other trivalent actinides/lanthanides provides additional qualitative support on the formation of these ternary complexes.

Curium fluorescence spectroscopy in the presence of calcium provided evidence for the formation of a never before reported ternary Ca–Cm(iii)–EDTA complex. While Cm(iii)–EDTA fluorescence spectroscopy was shown to be dependent on pH_m_, suggesting the formation of Cm(iii)–OH–EDTA complex(es), the addition of calcium to the system provided novel evidence for the formation of calcium-containing Cm(iii)–EDTA complexes. The use of calcium titrations at pH_m_ 7–12 and fluorescence lifetime calculations allowed for the identification of a ternary Ca–Cm(iii)–EDTA complex, which is expected to form at pH_m_ ≤11, and a potential additional quaternary Ca–Cm(iii)–OH–EDTA complex that forms under hyperalkaline conditions (pH_m_ ≈ 12). Further experimental studies are necessary to define the stoichiometries of these calcium-containing Cm–EDTA complexes. Finally, the formation of ternary Ca–Pu(iii)–EDTA and/or quaternary Ca–Pu(iii)–OH–EDTA complex(es) is strongly supported by solubility experiments conducted over a wide range of total calcium concentrations (1 mM ≤ [Ca(ii)]_tot_ ≤ 3.5 M) and theoretical model calculations. Solubility data exhibited a pH_m_-independent trend from pH_m_ ≈ 7.5–10 instead of the predicted decrease in *m*(Pu)_tot_ with increasing calcium concentration based on the available model in the literature. Finally, a comparison of aqueous plutonium oxidation state distribution in Pu(iii)–EDTA systems with and without calcium indicated that the presence of calcium may prevent the short-term (*t* ≤45 days) partial oxidation of Pu(iii) to Pu(iv) through the stabilization of Ca–Pu(iii)–EDTA species in solution.

It is expected that the formation of ternary/quaternary calcium-containing Pu(iii)–EDTA species will impact the previously defined Pu(iii)/Pu(iv) redox boundary, possibly increasing the stability field of Pu(iii)_aq_ species under conditions relevant to the context of nuclear waste disposal. Additional experimental efforts are needed to conclusively determine the stoichiometry and thermodynamic stability of Pu(iii)–OH–EDTA and Ca–Pu(iii)–EDTA complex(es) and their impact on the Pu(iii)/Pu(iv) redox boundary. The identification of these novel ternary and quaternary complexes for the An(iii)–EDTA–H_2_O and Ca–An(iii)–EDTA–H_2_O systems (An = Cm or Pu) is of great importance and future work must be considered for additional cations of environmental importance (*e.g.*, Mg or Fe) and their potential for ternary/quaternary complex formation with tri- and tetravalent actinides.

## Funding sources

This work was supported by the DOE Scholars Program (sponsored by the U.S. Department of Energy, administered by the Oak Ridge Institute for Science and Education, and funded by the WIPP project, DOE-CBFO) and the Institute for Nuclear Waste Disposal (Karlsruhe Institute of Technology). Open Access was funded by the KIT-Publication Fund of the Karlsruhe Institute of Technology.

## Conflicts of interest

There are no conflicts of interest to declare.

## Supplementary Material

RA-012-D1RA09010K-s001
