# Supplementary material for: Pu(iii) and Cm(iii) in the presence of EDTA: aqueous speciation, redox behavior, and the impact of Ca(ii)
Source: RSC Adv. 2022 Mar 25;12(15):9478–93. doi: 10.1039/d1ra09010k (PMC8985188; doi:10.1039/d1ra09010k)
Supplement: RA-012-D1RA09010K-s001 [file RA-012-D1RA09010K-s001.pdf]

Electronic Supplementary Material (ESI) for RSC Advances.
This journal is © The Royal Society of Chemistry 2022

Supporting Information

Pu(III) in the Presence of EDTA: Aqueous Speciation, Redox Behavior, and the Impact of Ca(II)

Nicole A. DiBlasi,[a,b]* Agost G. Tasi,[b] Michael Trumm,[b] Xavier Gaona,[b,]* David Fellhauer,[b] Andreas Schnurr,[b] Kathy Dardenne,[b] Jörg Rothe,[b] Donald T. Reed,[c] Amy E. Hixon,[a,]* Marcus Altmaier[b]

[a] Department of Civil & Environmental Engineering & Earth Sciences, University of Notre Dame, 301 Stinson-Remick, Notre Dame, IN 46556 USA; [b] Karlsruhe Institute of Technology, Institute for Nuclear Waste Disposal, P.O. Box 3640, 76021, Karlsruhe, Germany; [c] Los Alamos National Laboratory, 1400 University Dr., Carlsbad, NM 88220 USA

*corresponding authors: xavier.gaona@kit.edu (X. Gaona); ahixon@nd.edu (A. E. Hixon); nicole.a.diblasi@gmail.com (N. A. DiBlasi)

Table of Contents

$Pu(OH)_3$(am) Synthesis Procedure 3

Table S1. Selected equilibrium constants (log $K°$ or log $\beta°$) for PHREEQC thermodynamic calculations of Pu(III)-EDTA solubility and speciation. 4

Table S2. Ion interaction coefficients ($\varepsilon_{i,j}$) for PHREEQC and PHREEPLOT thermodynamic calculations. 5

Table S3. Experimental conditions of the $Pu(OH)_3$(am) undersaturation batch solubility samples in the presence of calcium and/or EDTA. 6

Table S4. Simulated WIPP brine formulation.[7] 6

Table S5. Pu L_{III}-edge XANES white line and inflection point energies (eV) determined in this work for Pu-EDTA-(Ca) aqueous phase samples or reported in the literature for Pu(III) and Pu(IV) references. 6

Table S6. Pu L_{III}-edge XANES white line energies (eV) determined in this work for solid phase Pu-EDTA samples or reported in the literature for Pu(IV) references. 7

Table S7. Average bond distances (Å) of DFT optimized Ca-Pu-EDTA complexes calculated at the hybrid density functional theory level (B3LYP). 7

Figure S1. Pourbaix diagram of the Pu-EDTA system at [Pu] = 1.1 mM, [EDTA] = 1 mM, I = 0.1 M, and 0 M $CaCl_2$ calculated from values in Tables S1 and S2. Experimental pH_m and pe measurements are shown for experiments conducted in the presence of Sn(II) (green diamonds) or in the presence of dithionite (DT; red triangles). Solid black lines correspond to redox borderlines between aqueous plutonium species and dashed black lines represent redox borderlines between solid plutonium species. 8

Figure S2. Pu L_{III}-edge XANES spectra measured *in-situ* for the aqueous phase of $Pu(OH)_3$(am) equilibrated with 1 mM EDTA and I = 0.1 M at pH_m = (7.6 ± 0.1) for 20 days. The spectra of the

references for $Pu(IV)_{aq}$ (red line) and $Pu(III)_{aq}$ (blue line) reported in Brendebach et al.[8] are shown for comparison. 9

Figure S3. Pourbaix diagram of the Pu-PO_4-EDTA system at [Pu] = 1.0 mM, [PO_4^{3-}] = 0.00032 M, [EDTA] = 0.0004 M, and I = 0.1 M calculated from values in Tables S1 and S2 with experimental pH_m and pe measurements from Rai et al.[4] Solid black lines correspond to redox borderlines between aqueous plutonium species and dashed black lines represent redox borderlines between solid plutonium compounds. Dotted lines correspond to the redox conditions for experiments in which hydroquinone (HQ, green) or dithionite (DT, pink) were used as redox buffers. 10

Figure S4. (a) Peak fitting results of laser fluorescence spectra of curium ([Cm] = 10^{-7} M) equilibrated in solutions containing $[EDTA]_{tot}$ = 1 mM and I = 0.1 as a function of pH_m. (b) Fraction of Cm-EDTA species calculated from peak fitting integrated peak areas as a function of pH_m. 11

Figure S5. Linear free energy relationship between the step-wise formation constants: log $K°_{111}$ for trivalent lanthanide- and actinide-EDTA complexes with OH^- ion derived within this work or adapted from the literature[10,11] as plotted against the effective ionic radii (Å) of the trivalent metal ions at a coordination number (CN) of 6. The gray dashed line represents the anticipated trend along the series. 12

Figure S6. Laser fluorescence spectra of curium ([Cm] = 10^{-7} M) equilibrated in solutions containing $[EDTA]_{tot}$ = 1 mM and I = 0.1 or 10.5 M as a function of calcium concentration (0 M ≤ [$CaCl_2$] ≤ 3.5 M) represented as either spectra with intensity normalized by the sum area or background corrected intensity: (a) pH_m 7 normalized spectra, (b) pH_m 9 normalized spectra, (c) pH_m 11 normalized spectra, (d) pH_m 12 normalized spectra, (e) pH_m 11 background corrected intensity spectra, and (f) pH_m 12 background corrected intensity spectra. Cm-EDTA peak positions are identified by black dashed vertical lines and Ca-Cm-EDTA peak positions are identified by colored dashed vertical lines. 13

Figure S7. Pu L_{III}-edge XANES spectra measured *in-situ* for $Pu(OH)_3$(am) equilibrated with dithionite (DT), 1 mM EDTA, 20 mM $CaCl_2$, and I = 0.1 M at pH_m = (9.0 ± 0.1) for 20 days. The spectra of the reference PuO_2(ncr,hyd) solid phase (red line) reported in Tasi et al.,[9] and freshly synthesized $Pu(OH)_3$(am) solid phase (blue line) are shown for comparison. 14

Figure S8. Pourbaix diagrams of the Pu-EDTA system at [Pu] = 1.1 mM, [EDTA] = 1 mM, I = 0.1 M, and (a) 1 mM $CaCl_2$ or (b) 20 mM $CaCl_2$ calculated from values in Tables S1 and S2. Experimental pH_m and pe measurements are shown for experiments conducted in the presence of Sn(II) (solid green diamonds), in the presence of dithionite (DT) with 0–20 mM $CaCl_2$ (solid red triangles), in the presence of DT with 3.5 M $CaCl_2$ (open red triangles), and in the absence of any redox buffer in the simulated WIPP brine (solid blue circles). Solid black lines correspond to redox borderlines between aqueous plutonium species and dashed black lines represent redox borderlines between solid plutonium compounds. 15

Figure S9. Pu L_{III} XANES spectra measured *in-situ* for the aqueous phase of $Pu(OH)_3$(am) equilibrated with 1 mM EDTA, 1 mM $CaCl_2$, and I = 0.1 M for 20 days. The spectra of the references for $Pu(IV)_{aq}$ (red lines) and $Pu(III)_{aq}$ (blue lines) reported in Brendebach et al.[8] are shown for comparison. 16

References 17

$Pu(OH)_3$(am) Synthesis Procedure

A Pu(IV) stock solution in 2 M $HClO_4$ was reduced electrochemically with a platinized Pt-wire working electrode to $Pu(III)_{aq}$ by applying a potential range of –0.20 to –0.40 V (*vs.* SHE) with the use of an Ag/AgCl/3 M NaCl reference electrode (Metrohm) and a standard Pt electrode acting as counter electrode. The reference and counter electrodes were separated from the plutonium stock by ceramic diaphragms and Teflon capillaries filled with 1.7 M $HClO_4$ solutions attached to quartz tubes. The three electrodes were connected to a potentiostat (Princeton Applied Research Model 362) to control constant voltage. To avoid overheating, the current never exceeded 10 mA. The procedure was monitored by means of visible-near infrared (Vis-NIR) absorption spectroscopy on a single-beam diode array photometer (λ = 400–1020 nm, Carl Zeiss AG, MSC-501). The electrochemically-prepared Pu(III) stock solution was precipitated as $Pu(OH)_3$(am) by its slow addition to a 0.1 M TRIS buffer solution (pH_m = 9.47) with 5 mM $Na_2S_2O_4$ redox buffering agent present. Following precipitation, the solid phase was washed with and subsequently suspended in a pH 11 NaOH solution. The resulting solid phase was used as the solubility-controlling solid phase in the undersaturation solubility studies in the presence of EDTA and calcium and as the reference material in the X-ray absorption near edge structure (XANES) study.

Table S1. Selected equilibrium constants (log $K°$ or log $\beta°$) for PHREEQC thermodynamic calculations of Pu(III)-EDTA solubility and speciation.

Reaction	log $K°$ or log $\beta°$	Reference
$H_4(EDTA)(cr) \Leftrightarrow EDTA^{4-} + 4H^+$	-(27.220 ± 0.200)	1
$EDTA^{4-} + H^+ \Leftrightarrow H(EDTA)^{3-}$	(11.240 ± 0.030)	1
$EDTA^{4-} + 2H^+ \Leftrightarrow H_2(EDTA)^{2-}$	(18.040 ± 0.036)	1
$EDTA^{4-} + 3H^+ \Leftrightarrow H_3(EDTA)^-$	(21.190 ± 0.062)	1
$EDTA^{4-} + 4H^+ \Leftrightarrow H_4(EDTA)(aq)$	(23.420 ± 0.200)	1
$EDTA^{4-} + 5H^+ \Leftrightarrow H_5(EDTA)^+$	(24.720 ± 0.223)	1
$EDTA^{4-} + 6H^+ \Leftrightarrow H_6(EDTA)^{2+}$	(24.220 ± 0.300)	1
$PO_4^{3-} + H^+ \Leftrightarrow HPO_4^{2-}$	(12.350 ± 0.030)	2
$PO_4^{3-} + 2H^+ \Leftrightarrow H_2PO_4^-$	(19.562 ± 0.033)	2
$PO_4^{3-} + 3H^+ \Leftrightarrow H_3PO_4(aq)$	(21.702 ± 0.044)	2
$2H_3PO_4(aq) \Leftrightarrow H_4P_2O_7(aq) + H_2O$	-(2.790 ± 0.170)	2
$Ca^{2+} + EDTA^{4-} \Leftrightarrow Ca(EDTA)^{2-}$	(12.690 ± 0.060)	1
$Ca^{2+} + EDTA^{4-} + H^+ \Leftrightarrow Ca(HEDTA)^-$	(16.230 ± 0.108)	1
$Na^+ + EDTA^{4-} \Leftrightarrow Na(EDTA)^{3-}$	(2.800 ± 0.200)	1
$Pu(OH)_3(am) \Leftrightarrow Pu^{3+} + 3OH^-$	-(27.47 ± 0.50)	3
$PuPO_4(cr,hyd) \Leftrightarrow Pu^{3+} + PO_4^{3-}$	-(24.42 ± 0.38)	4 Pitzer
$PuPO_4(cr,hyd) \Leftrightarrow Pu^{3+} + PO_4^{3-}$	-(24.28 ± 0.35)	4 SIT
$Pu^{3+} + H_2O \Leftrightarrow Pu(OH)^{2+} + H^+$	-(6.900 ± 0.300)	2
$Pu^{3+} + 2H_2O \Leftrightarrow Pu(OH)_2^+ + 2H^+$	-(15.100 ± 0.300)	2
$Pu^{3+} + 3H_2O \Leftrightarrow Pu(OH)_3(aq) + 3H^+$	-(26.200 ± 0.300)	2
$Pu^{3+} + PO_4^{3-} \Leftrightarrow PuPO_4(aq)$	≤ 14	4
$Pu^{3+} + H_2PO_4^- \Leftrightarrow Pu(H_2PO_4)^{2+}$	(2.200 ± 0.060)	2
$Pu^{3+} + EDTA^{4-} \Leftrightarrow Pu(EDTA)^-$	(20.180 ± 0.370)	1
$Pu^{3+} + EDTA^{4-} + H^+ \Leftrightarrow Pu(HEDTA)(aq)$	(22.020 ±0.260)	1
$Pu^{3+} + EDTA^{4-} \Leftrightarrow Pu(EDTA)^-$	(20.15 ± 0.59)	4 Pitzer
$Pu^{3+} + EDTA^{4-} \Leftrightarrow Pu(EDTA)^-$	(19.97 ± 0.62)	4 SIT
$PuO_2 \bullet 2H_2O(am,hyd) \Leftrightarrow Pu^{4+} + 4OH^-$	-(58.33 ± 0.52)	5
$Pu^{4+} + H_2O \Leftrightarrow Pu(OH)^{3+} + H^+$	(0.600 ± 0.200)	2
$Pu^{4+} + 2H_2O \Leftrightarrow Pu(OH)_2^{2+} + 2H^+$	(0.600 ± 0.300)	2
$Pu^{4+} + 3H_2O \Leftrightarrow Pu(OH)_3^+ + 3H^+$	-(2.300 ± 0.400)	2
$Pu^{4+} + 4H_2O \Leftrightarrow Pu(OH)_4(aq) + 4H^+$	-(8.500 ± 0.500)	2
$Pu^{4+} + EDTA^{4-} + H_2O \Leftrightarrow Pu(OH)(EDTA)^- + H^+$	(23.00 ± 0.30)	6
$Pu^{4+} + EDTA^{4-} + 2H_2O \Leftrightarrow Pu(OH)_2(EDTA)^{2-} + 2H^+$	(18.02 ± 0.30)	6
$Pu^{4+} + EDTA^{4-} + 3H_2O \Leftrightarrow Pu(OH)_3(EDTA)^{3-} + 3H^+$	(8.50 ± 0.30)	6
$Ca^{4+} + Pu^{4+} + EDTA^{4-} + 4H_2O \Leftrightarrow CaPu(OH)_4(EDTA)^{2-} + 4H^+$	(8.90 ± 0.10)*	6

*Stoichiometry and formation constant of quaternary Ca-Pu(IV)-OH-EDTA complex are considered tentative, as discussed in DiBlasi et al.[6]

Table S2. Ion interaction coefficients ($\varepsilon_{i,j}$) for PHREEQC and PHREEPLOT thermodynamic calculations.

i	*j*	$\varepsilon_{i,j}$ (kg·mol^{-1})
H^+	Cl^-	0.12
Cl^-	Na^+	0.03
OH^-	Na^+	0.04
PO_4^{3-}	Na^+	-0.25
HPO_4^{2-}	Na^+	-0.15
$H_2PO_4^-$	Na^+	-0.08
Ca^{2+}	Cl^-	0.14
$EDTA^{4-}$	Na^+	0.32
$H(EDTA)^{3-}$	Na^+	-0.1
$H_2(EDTA)^{2-}$	Na^+	-0.37
$H_3(EDTA)^-$	Na^+	-0.33
$H_5(EDTA)^+$	Cl^-	-0.23
$H_6(EDTA)^{2+}$	Cl^-	-0.2
Pu^{3+}	Cl^-	0.23
$Pu(OH)^{2+}$	Cl^-	-0.04
$Pu(OH)_2^+$	Cl^-	-0.06
$PuH_2PO_4^{2+}$	Cl^-	0.00
$Pu(EDTA)^-$	Na^+	-0.33
Pu^{4+}	Cl^-	0.40
$Pu(OH)^{3+}$	Cl^-	0.2
$Pu(OH)_2^{2+}$	Cl^-	0.1
$Pu(OH)_3^+$	Cl^-	0.05
$PuCl^{3+}$	Cl^-	0.1
$Pu(OH)(EDTA)^-$	Na^+	-0.33
$Pu(OH)_2(EDTA)^{2-}$	Na^+	-0.37
$Pu(OH)_3(EDTA)^{3-}$	Na^+	-0.1
$CaPu(OH)_4(EDTA)^{2-}$	Na^+	-0.1

Table S3. Experimental conditions of the $Pu(OH)_3$(am) undersaturation batch solubility samples in the presence of calcium and/or EDTA.

$Pu(OH)_3$(am) (mg)	EDTA (M)	pH_m	$CaCl_2$ (M)	I (M)	Redox Buffer*	Equilibration Time (days)
1.5 ± 0.1	$(1.00 \pm 0.01)\cdot10^{-3}$	7.6 ± 0.1	0	0.10 ± 0.01	DT	45
1.5 ± 0.1	$(1.00 \pm 0.01)\cdot10^{-3}$	7.3 ± 0.1	0	0.10 ± 0.01	Sn(II)	45
1.5 ± 0.1	$(1.00 \pm 0.01)\cdot10^{-3}$	7.8 ± 0.7	$(1.00 \pm 0.01)\cdot10^{-3}$	0.10 ± 0.01	DT	45
1.5 ± 0.1	$(1.00 \pm 0.01)\cdot10^{-3}$	9.6 ± 0.2	$(1.00 \pm 0.01)\cdot10^{-3}$	0.10 ± 0.01	Sn(II)	45
1.5 ± 0.1	$(1.00 \pm 0.01)\cdot10^{-3}$	8.2 ± 0.3	$(2.00 \pm 0.02)\cdot10^{-2}$	0.10 ± 0.01	DT	45
1.5 ± 0.1	$(1.00 \pm 0.01)\cdot10^{-3}$	9.2 ± 0.1	$(2.00 \pm 0.02)\cdot10^{-2}$	0.10 ± 0.01	Sn(II)	45
1.5 ± 0.1	$(1.00 \pm 0.01)\cdot10^{-3}$	8.3 ± 0.1	3.50 ± 0.01	10.50 ± 0.03	DT	45
1.5 ± 0.1	$(1.00 \pm 0.01)\cdot10^{-3}$	9.7 ± 0.1	WIPP Simulated Brine (90%)		---	45

*DT = (2.0 ± 0.1) mM sodium dithionite; Sn(II) = (0.5 ± 0.1) mM $SnCl_2$

Table S4. Simulated WIPP brine formulation.[7]

Brine (Formulation)	Reagent ($g\cdot L^{-1}$)								Calc. Ionic Strength (M)
	$Na_2B_4O_7$• $10H_2O$	$MgCl_2$• $6H_2O$	$CaCl_2$• $2H_2O$	LiCl	KCl	NaBr	Na_2SO_4	NaCl	
90%, pH_m* = 9 Simulated Brine	13.36	188.46	1.787	0.143	30.73	2.176	22.50	151.54	6.40

*quoted in the original publication as pC_{H^+}

Table S5. Pu L_{III}-edge XANES white line and inflection point energies (eV) determined in this work for Pu-EDTA-(Ca) aqueous phase samples or reported in the literature for Pu(III) and Pu(IV) references.

Sample Name	White Line (eV)	ΔE between Pu Fermi Level E_0 and WL (eV)	ΔE between WL and First Feature (eV)
$Pu(III)_{aq}$ [a]	18062.6	5.6 ± 0.5	34.8 ± 0.5
DT, 0 mM $CaCl_2$, pH_m = 7.6	18064.6	7.6 ± 0.5	36.3 ± 0.5
DT, 1 mM $CaCl_2$, pH_m = 9	18062.6	5.6 ± 0.5	33.7 ± 0.5
$Pu(IV)_{aq}$ [a]	18067.6	10.6 ± 0.5	38.1 ± 0.5

[a] from Brendebach et al.[8]

Table S6. Pu L_{III}-edge XANES white line energies (eV) determined in this work for solid phase Pu-EDTA samples or reported in the literature for Pu(IV) references.

Sample Name	White Line (eV)	ΔE between Pu Fermi Level E_0 and WL (eV)	ΔE between WL and First Feature (eV)
$Pu(OH)_3$(am)	18062.1	5.1 ± 0.5	34.2 ± 0.5
DT, 20 mM $CaCl_2$, pH_m = 9, solid	18062.6	5.6 ± 0.5	33.7 ± 0.5
PuO_2(ncr,hyd)[a]	18067.6	10.6 ± 0.5	38.1 ± 0.5

[a] from Tasi et al.[9]

Table S7. Average bond distances (Å) of DFT optimized Ca-Pu-EDTA complexes calculated at the hybrid density functional theory level (B3LYP).

Complex	d(Pu–O_{EDTA})	d(Pu–N_{EDTA})	d(Pu–O_{H2O})	d(Pu–O_{OH})
$[(Pu(EDTA)(H_2O)_2)Ca(H_2O)_4]^{+}$	2.47	2.72	2.64	–
$[(Pu(OH)_2(EDTA))Ca_3(H_2O)_{12}]^{3+}$	2.52	2.90	–	2.44
$[(Pu(OH)(EDTA)H_2O)Ca_2(H_2O)_8]^{2+}$	2.52	2.87	2.60	2.32

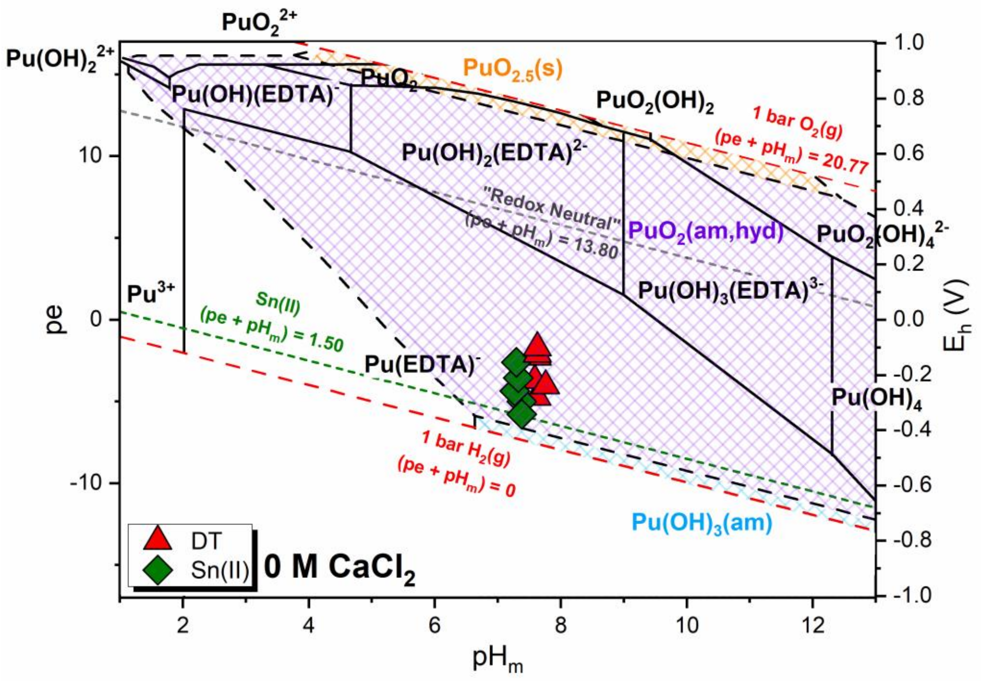

Figure S1. Pourbaix diagram of the Pu-EDTA system at [Pu] = 1.1 mM, [EDTA] = 1 mM, I = 0.1 M, and 0 M $CaCl_2$ calculated from values in Tables S1 and S2. Experimental pH_m and pe measurements are shown for experiments conducted in the presence of Sn(II) (green diamonds) or in the presence of dithionite (DT; red triangles). Solid black lines correspond to redox borderlines between aqueous plutonium species and dashed black lines represent redox borderlines between solid plutonium species.

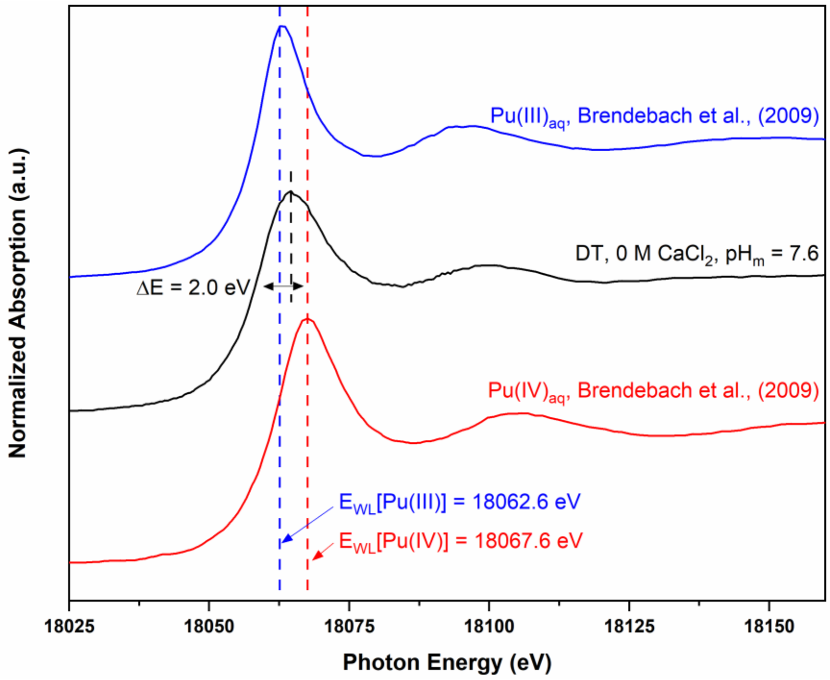

Figure S2. Pu L_{III}-edge XANES spectra measured *in-situ* for the aqueous phase of $Pu(OH)_3$(am) equilibrated with 1 mM EDTA and I = 0.1 M at pH_m = (7.6 ± 0.1) for 20 days. The spectra of the references for $Pu(IV)_{aq}$ (red line) and $Pu(III)_{aq}$ (blue line) reported in Brendebach et al.[8] are shown for comparison.

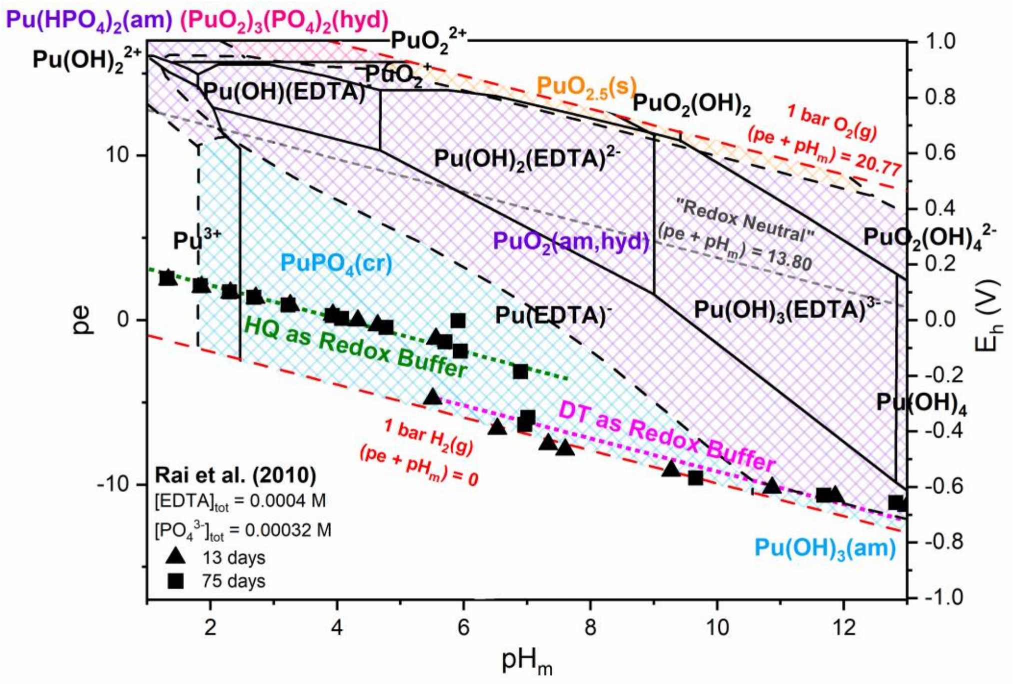

Figure S3. Pourbaix diagram of the Pu-PO_4-EDTA system at [Pu] = 1.0 mM, [PO_4^{3-}] = 0.00032 M, [EDTA] = 0.0004 M, and *I* = 0.1 M calculated from values in Tables S1 and S2 with experimental pH_m and pe measurements from Rai et al.[4] Solid black lines correspond to redox borderlines between aqueous plutonium species and dashed black lines represent redox borderlines between solid plutonium compounds. Dotted lines correspond to the redox conditions for experiments in which hydroquinone (HQ, green) or dithionite (DT, pink) were used as redox buffers.

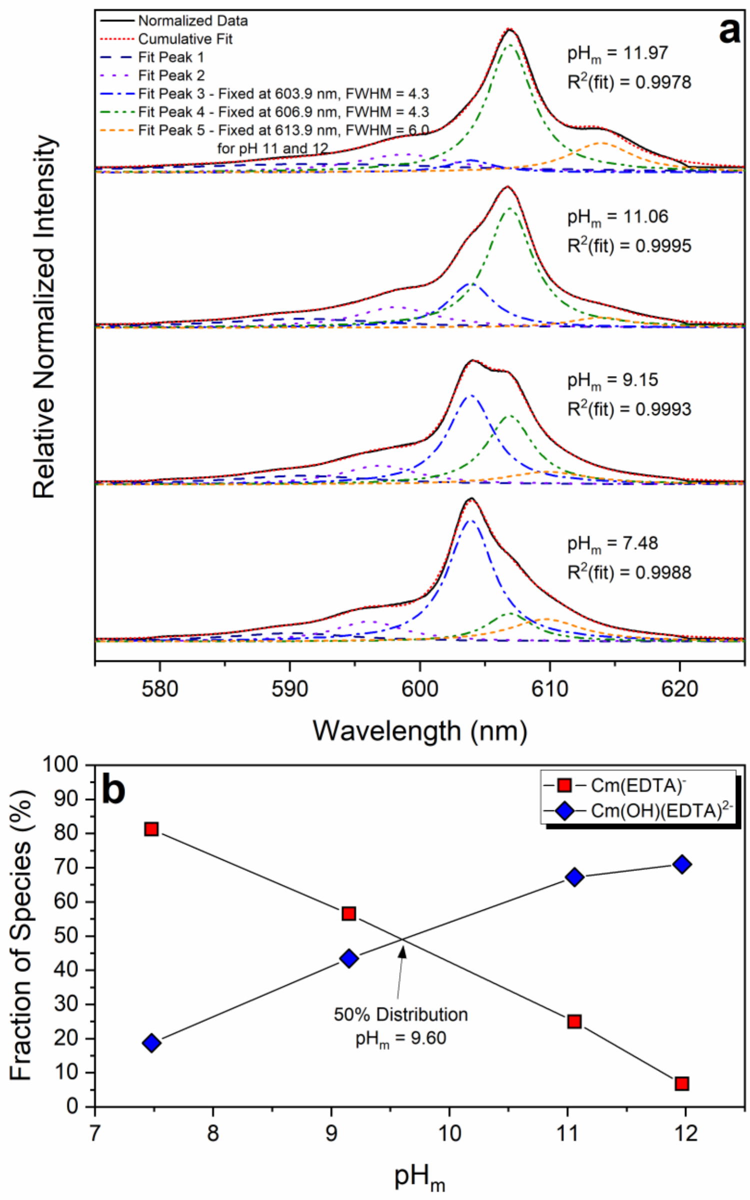

Figure S4. (a) Peak fitting results of laser fluorescence spectra of curium ([Cm] = 10^{-7} M) equilibrated in solutions containing $[EDTA]_{tot}$ = 1 mM and I = 0.1 as a function of pH_m. (b) Fraction of Cm-EDTA species calculated from peak fitting integrated peak areas as a function of pH_m.

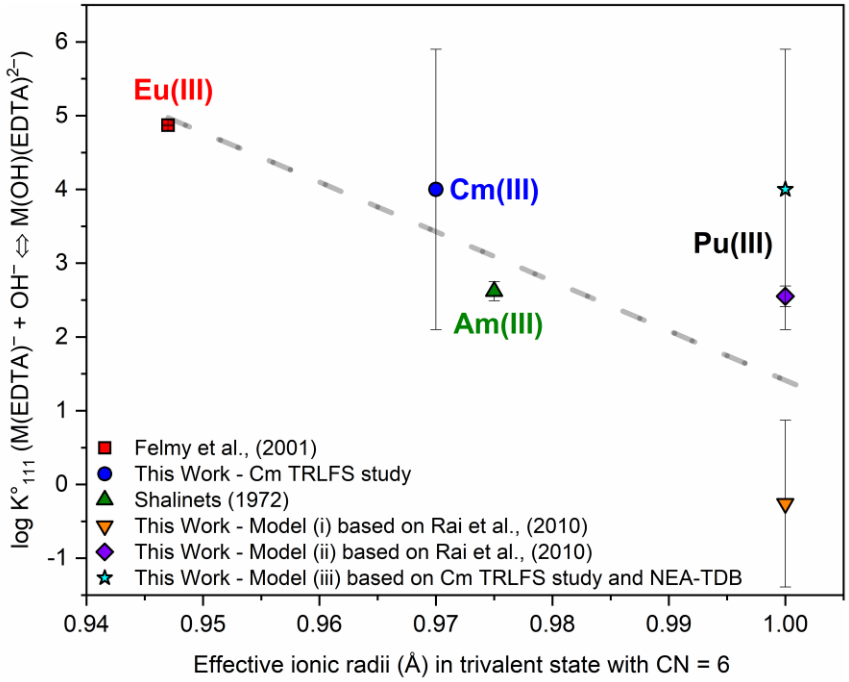

Figure S5. Linear free energy relationship between the step-wise formation constants: log $K°_{111}$ for trivalent lanthanide- and actinide-EDTA complexes with OH^- ion derived within this work or adapted from the literature[10,11] as plotted against the effective ionic radii (Å) of the trivalent metal ions at a coordination number (CN) of 6. The gray dashed line represents the anticipated trend along the series.

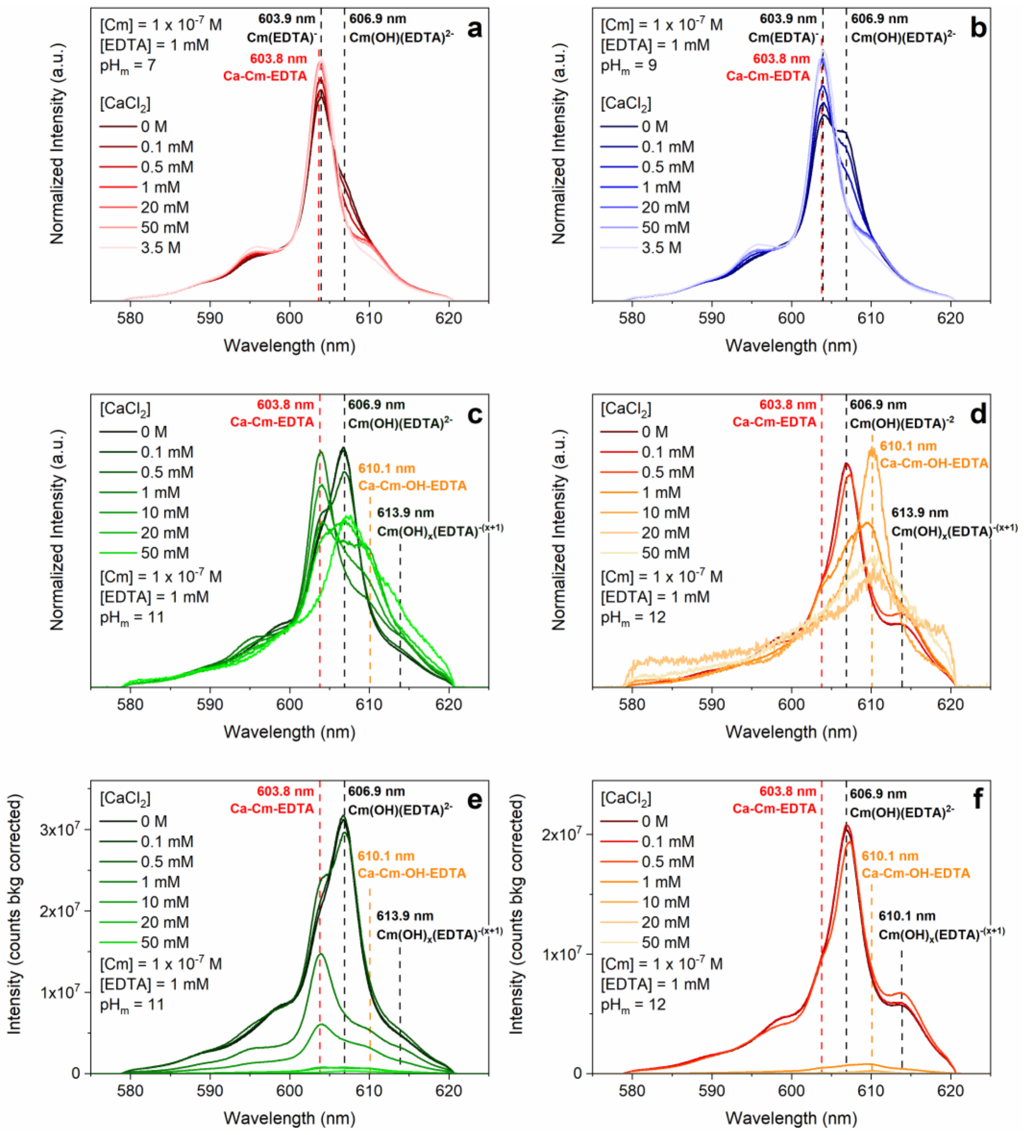

Figure S6. Laser fluorescence spectra of curium ([Cm] = 10^{-7} M) equilibrated in solutions containing $[EDTA]_{tot}$ = 1 mM and I = 0.1 or 10.5 M as a function of calcium concentration (0 M ≤ $[CaCl_2]$ ≤ 3.5 M) represented as either spectra with intensity normalized by the sum area or background corrected intensity: (a) pH_m 7 normalized spectra, (b) pH_m 9 normalized spectra, (c) pH_m 11 normalized spectra, (d) pH_m 12 normalized spectra, (e) pH_m 11 background corrected intensity spectra, and (f) pH_m 12 background corrected intensity spectra. Cm-EDTA peak positions are identified by black dashed vertical lines and Ca-Cm-EDTA peak positions are identified by colored dashed vertical lines.

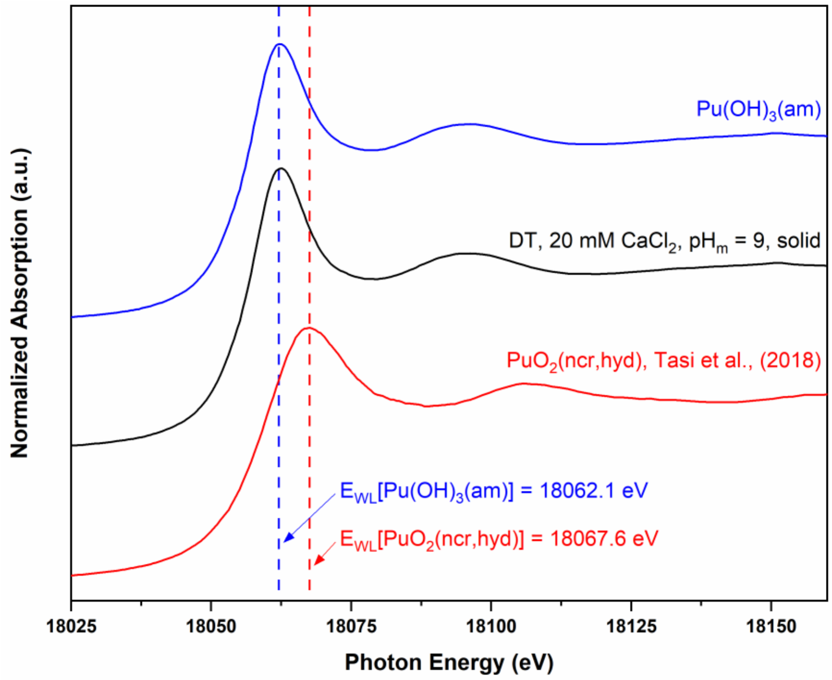

Figure S7. Pu L_{III}-edge XANES spectra measured *in-situ* for $Pu(OH)_3$(am) equilibrated with dithionite (DT), 1 mM EDTA, 20 mM $CaCl_2$, and I = 0.1 M at pH_m = (9.0 ± 0.1) for 20 days. The spectra of the reference PuO_2(ncr,hyd) solid phase (red line) reported in Tasi et al.,[9] and freshly synthesized $Pu(OH)_3$(am) solid phase (blue line) are shown for comparison.

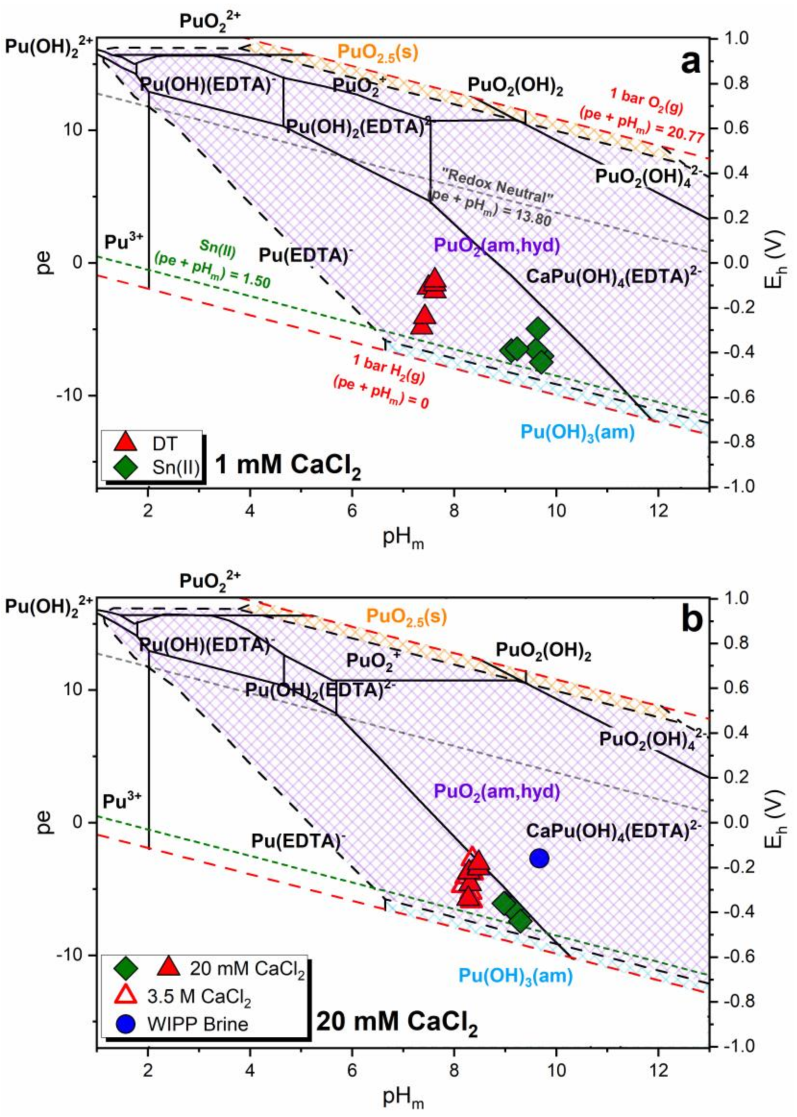

Figure S8. Pourbaix diagrams of the Pu-EDTA system at [Pu] = 1.1 mM, [EDTA] = 1 mM, I = 0.1 M, and (a) 1 mM $CaCl_2$ or (b) 20 mM $CaCl_2$ calculated from values in Tables S1 and S2. Experimental pH_m and pe measurements are shown for experiments conducted in the presence of Sn(II) (solid green diamonds), in the presence of dithionite (DT) with 0–20 mM $CaCl_2$ (solid red triangles), in the presence of DT with 3.5 M $CaCl_2$ (open red triangles), and in the absence of any redox buffer in the simulated WIPP brine (solid blue circles). Solid black lines correspond to redox borderlines between aqueous plutonium species and dashed black lines represent redox borderlines between solid plutonium compounds.

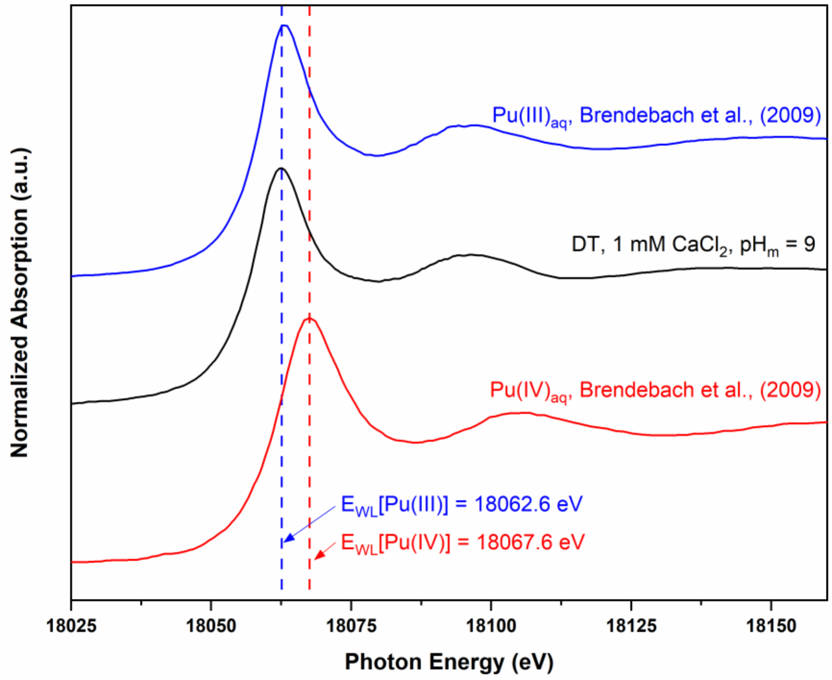

Figure S9. Pu L_{III} XANES spectra measured *in-situ* for the aqueous phase of $Pu(OH)_3(am)$ equilibrated with 1 mM EDTA, 1 mM $CaCl_2$, and I = 0.1 M for 20 days. The spectra of the references for $Pu(IV)_{aq}$ (red lines) and $Pu(III)_{aq}$ (blue lines) reported in Brendebach et al.[8] are shown for comparison.

References

1 W. Hummel, G. Anderegg, I. Puigdomènech, L. Rao and O. Tochiyama, *Chemical Thermodynamics of Compounds and Complexes of U, Np, Pu, Am, Tc, Se, Ni and Zr with Selected Organic Ligands*, OECD Nuclear Energy Agency, Issy-les-Moulineaux, France, 2005, vol. 9.

2 I. Grenthe, X. Gaona, A. V. Plyasunov, L. Rao, W. H. Runde, B. Grambow, R. J. M. Konings, A. L. Smith and E. E. Moore, *Second Update on the Chemical Thermodynamics of Uranium, Neptunium, Plutonium, Americium and Technetium*, OECD Nuclear Energy Agency, Boulogne-Billancourt, France, 2020, vol. 14.

3 H.-R. Cho, Y.-S. Youn, E. Chang Jung and W. Cha, *Dalton Trans.*, 2016, **45**, 19449–19457.

4 D. Rai, D. A. Moore, A. R. Felmy, K. M. Rosso and H. Bolton, *J. Solution Chem.*, 2010, **39**, 778–807.

5 V. Neck, M. Altmaier and T. Fanghänel, *C.R. Chim.*, 2007, **10**, 959–977.

6 N. A. DiBlasi, A. G. Tasi, X. Gaona, D. Fellhauer, K. Dardenne, J. Rothe, D. T. Reed, A. E. Hixon and M. Altmaier, *Sci. Total Environ.*, 2021, **783**, 146993.

7 J. F. Lucchini, D. Cleveland, L. Smith, D. T. Reed and N. Elkins, *Brine Preparation*, 2014.

8 B. Brendebach, N. L. Banik, C. M. Marquardt, J. Rothe, M. Denecke and H. Geckeis, *Radiochim. Acta*, 2009, **97**, 701–708.

9 A. Tasi, X. Gaona, D. Fellhauer, M. Böttle, J. Rothe, K. Dardenne, D. Schild, M. Grivé, E. Colàs, J. Bruno, K. Källström, M. Altmaier and H. Geckeis, *Radiochim. Acta*, 2018, **106**, 259–279.

10 A. R. Felmy, Z. Wang, D. A. Dixon, A. G. Joly, J. R. Rustad and M. J. Mason, in *Nuclear Site Remediation*, American Chemical Society, 2001, vol. 778, pp. 63–82.

11 A. B. Shalinets, *Sov. Radiochem.*, 1972, **14**, 285–289.
